# Additive Manufacturing of α-Amino Acid Based Poly(ester amide)s for Biomedical Applications

**DOI:** 10.1021/acs.biomac.1c01417

**Published:** 2022-01-20

**Authors:** Vahid Ansari, Andrea Calore, Jip Zonderland, Jules A. W. Harings, Lorenzo Moroni, Katrien V. Bernaerts

**Affiliations:** † Complex Tissue Regeneration Department, MERLN Institute for Technology Inspired Regenerative Medicine, Maastricht University, Universiteitssingel 40, 6229 ER Maastricht, The Netherlands; ‡ Aachen-Maastricht Institute for Biobased Materials (AMIBM), Maastricht University, P.O. Box 616, 6200 MD Maastricht, The Netherlands

## Abstract

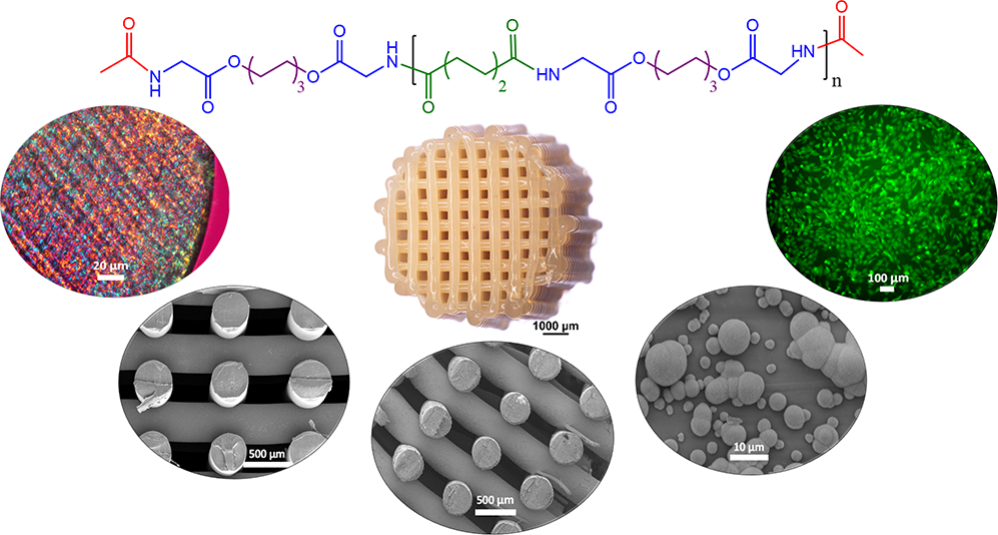

α-Amino acid based polyester amides (PEAs) are promising candidates for additive manufacturing (AM), as they unite the flexibility and degradability of polyesters and good thermomechanical properties of polyamides in one structure. Introducing α-amino acids in the PEA structure brings additional advantages such as (i) good cytocompatibility and biodegradability, (ii) providing strong amide bonds, enhancing the hydrogen-bonding network, (iii) the introduction of pendant reactive functional groups, and (iv) providing good cell–polymer interactions. However, the application of α-amino acid based PEAs for AM via fused deposition modeling (FDM), an important manufacturing technique with unique processing characteristics and requirements, is still lacking. With the aim to exploit the combination of these advantages in the creation, design, and function of additively manufactured scaffolds using FDM, we report the structure–function relationship of a series of α-amino acid based PEAs. The PEAs with three different molecular weights were synthesized via the active solution polycondensation, and their performance for AM applications was studied in comparison with a commercial biomedical grade copolymer of l-lactide and glycolide (PLGA). The PEAs, in addition to good thermal stability, showed semicrystalline behavior with proper mechanical properties, which were different depending on their molecular weight and crystallinity. They showed more ductility due to their lower glass transition temperature (*T*_g_; 18–20 °C) compared with PLGA (57 °C). The rheology studies revealed that the end-capping of PEAs is of high importance for preventing cross-linking and further polymerization during the melt extrusion and for the steadiness and reproducibility of FDM. Furthermore, our data regarding the steady 3D printing performance, good polymer–cell interactions, and low cytotoxicity suggest that α-amino acid based PEAs can be introduced as favorable polymers for future AM applications in tissue engineering. In addition, their ability for formation of bonelike apatite in the simulated body fluid (SBF) indicates their potential for bone tissue engineering applications.

## Introduction

During the past few decades, additive manufacturing (AM) has attracted considerable attention from researchers around the world. Since AM has shown to be a versatile and promising fabrication technique for different applications, a lot of time and resources have been spent on improving the quality of AM products.^1−3^ Many AM techniques with high accuracy and complexity have been developed for the manufacture of various products, ranging from commercial goods and industrial pieces, to purposefully designed structures for art, design, and 3D scaffolds for biomedical uses.^1,4^ There are many factors, such as material properties, type of printer, and processing parameters, that can influence the AM procedure.^1,2,4,5^ To date, the search for materials such as metals, ceramics, and polymers that possess the technical requirements for a particular application remains an essential challenge in many interdisciplinary fields of science and industry related to AM.^2,3^

Polymer-based AM has attracted much attention due to the fact that polymers are the feed supplied in most types of 3D printers.^6^ Depending on the targeted application of an AM structure, various polymers with different chemical structures and physical properties can be exploited to be deposited via different AM techniques. Currently, polymer degradability is one of the key parameters for polymeric products being made for several applications, especially in the biomedical field. Different biodegradable polyesters, such as polycaprolactone (PCL),^7−9^ polylactide (PLA),^10−13^ and poly(lactic-*co*-glycolic acid) (PLGA)^14−16^ or a combination of these polymers,^16−19^ have been used in polymer-based AM. PLA has been commonly used in ultimately FDA approved biodegradable applications and, therefore, is well studied in the biomedical area. However, thermoplastic polyesters such as PLA and PLGA suffer from particular limitations, among which the most pronounced is thermal instability during melt processing technologies like extrusion-based AM.^20,21^ In the absence of stabilizers, which are often incompatible with FDA requirements and the human body, the ester bonds of these biomaterials are susceptible to chemical reactions like hydrolysis above their melting point, and consequently, the hydrolytic degradation rate can increase in the presence of moisture.^22,23^ Therefore, along with using the commercially available polymers, researchers are aware of the need for finding innovative materials with enhanced properties that could be introduced as new resources to the field of AM.

One of the key challenges in AM with thermoplastics has been finding polymers that not only possess thermally stable behavior during the processing conditions, but also provide sufficient mechanical strength for the aimed application. In order to obtain a polymeric macroscopic structure with the necessary properties for a particular application, the synthesis of a tailor-made polymeric design is needed. The advantage of synthetic thermoplastic polymers is that selecting the right building blocks in the polymer’s backbone enables the realization of tailored polymeric materials with controlled physical properties that optimally suit particular applications. Challenges in the use of synthetic polymers for medical applications, for example, include poor biocompatibility, toxic degradation and loss of mechanical properties through degradation.^24^ Via different methodological strategies, researchers have been working on commercially available materials to enhance the mechanical properties of the fabricated 3D structures. These methods consist of different polymer compositions for better printing performances^25^ or 3D printing of polymer composites instead of pure polymers.^26^

α-Amino acid based poly(ester amide)s (PEAs) seem promising candidates for polymer-based AM due to a unique combination of properties. They unite the biodegradability, biocompatibility, and flexibility of polyesters, and the thermomechanical stability of polyamides in one material, which can be finely tuned during chemical design and synthesis. Amide bonds are thermally and chemically more stable and also less susceptible to hydrolysis reactions than the ester bonds due to their higher double bond characteristic and lower electrophilicity of the carbonyl group.^27,28^ Therefore, PEAs are expected to improve the thermal stability upon melt processing, which is today a recurring problem in AM of thermoplastic biomaterials based on polyesters such as PLA. In addition, by embedding amino acids with functional groups capable of doing successive chemical reactions, in or aside the polymer’s backbone, researchers can induce chemical modifications such as attaching biologically favored pendant groups after AM.^29^

The use of α-amino acids in the PEA backbone may not only improve the biocompatibility of the resulting polymers, as the effective acidity of the degradation products is inferior in comparison to the hydroxy acids of degraded polyesters, but can also provide chemical solutions to improved, tailored, and tissue-specific biocompatibilization.^30,31^ Synthesizing α-amino acid based PEAs via the active solution polycondensation method provides PEAs, including naturally occurring building blocks (α-amino acids, diols, and dicarboxylic acids), with suitable biocompatibility and biodegradability for biomedical applications.^30,32−34^ Electrospinning and AM are important manufacturing techniques, which are being used for the fabrication of 3D structures for biomedical applications. Electrospinning of α-amino acid based PEA polymer solutions has been used for the fabrication of 3D structures for different biomedical applications.^35−39^ Gloria et al. have reported the use of α-amino acid based PEA blends (up to 20%, w/w) with poly(ε-caprolactone) (PCL) for 3D printing of scaffolds leading to increased hydrophilicity, improved cell adhesion and proliferation, and enhanced mechanical performance of the blend scaffolds in comparison with PCL.^40^ Nevertheless, using α-amino acid based PEAs as thermoplastic resources for FDM-based AM applications is still lacking. Beyond the promising properties from the biocompatibility and biodegradation perspectives, there are other important factors such as thermal stability, melt rheology behavior, mechanical properties, and polymer–cell interactions that need to be evaluated for α-amino acid based PEAs.

The current work describes the synthesis of α-amino acid based PEAs via the active solution polycondensation method and the evaluation of their capability for the fabrication of AM scaffolds. In order to study the effect of molecular weight on the printability of PEAs, different molecular weights were synthesized and subsequently end-capped. The rheological and thermal behavior were studied in order to gain knowledge about the printability window of the synthesized polymers. The mechanical properties of the PEAs were studied in terms of tensile strength, and compression tests were used to compare bulk and AM specimens. The structure of the AM scaffolds was also studied by scanning electron microscopy (SEM). Along with the thermal stability, rheological behavior, and mechanical properties of the PEA films and bulk specimens, their cell–polymer interactions and bioactivity in the simulated body fluid (SBF) were studied and compared with a commercial semicrystalline biomedical grade poly(lactide-*co*-glycolide) (PLGA).

## Experimental Section

### Materials

Adipoyl chloride (98%), acetic anhydride (99%), sodium chloride (99.5%), sodium bicarbonate (99.5%), sodium phosphate dibasic dehydrate (99%), calcium chloride (95%), and dry dimethylsolfoxide (DMSO; >99.9%) were purchased from Sigma-Aldrich. Glycine (>99%), *p*-toluenesulfonic acid monohydrate (pTsOH·H_2_O) (99%), 1,6-hexanediol (97%), *p*-nitrophenol (99%), calcium hydride (93%), and 4 Å molecular sieves were purchased from ACROS Organics. Triethylamine (>99%) was purchased from Fischer Scientific. Anhydrous calcium chloride (96%) was purchased from VWR. Acetone (99.8%), toluene (99.7%), ethyl acetate (99.8%), acetonitrile (99.9%), diethyl ether (99.8%), and methanol (99.9%) were purchased from Biosolve. Deuterated DMSO (DMSO-*d*_6_) and *N*,*N*-dimethylformamide (DMF-*d*_7_) were purchased from Cambridge Isotope Laboratories. Acetone was dried over anhydrous calcium chloride at room temperature, and toluene was dried with 4 Å molecular sieves before use. Triethylamine was dried through reflux over calcium hydride. A commonly used biomedical grade copolymer of l-lactide and glycolide in an 82/18 molar ratio, PLG 8218, was kindly provided by Corbion (The Netherlands) and used as reference material. Proteinaze K from Tritirachium album (lyophilized powder, BioUltra, ≥30 units/mg protein) was purchased from Sigma-Aldrich. CDP-Star Substrate (0.25 mM ready-to-use), CyQUANT Cell Proliferation Assay, and LDH Cytotoxicity Assay Kit were purchased from Thermo Fisher Scientific. All of the other materials were used as received without further purification.

### Synthesis of Monomers and Poly(ester amide)s (PEAs)

#### Synthesis of Di-*p*-nitrophenyl Adipate (Monomer A)

**Scheme 1:**
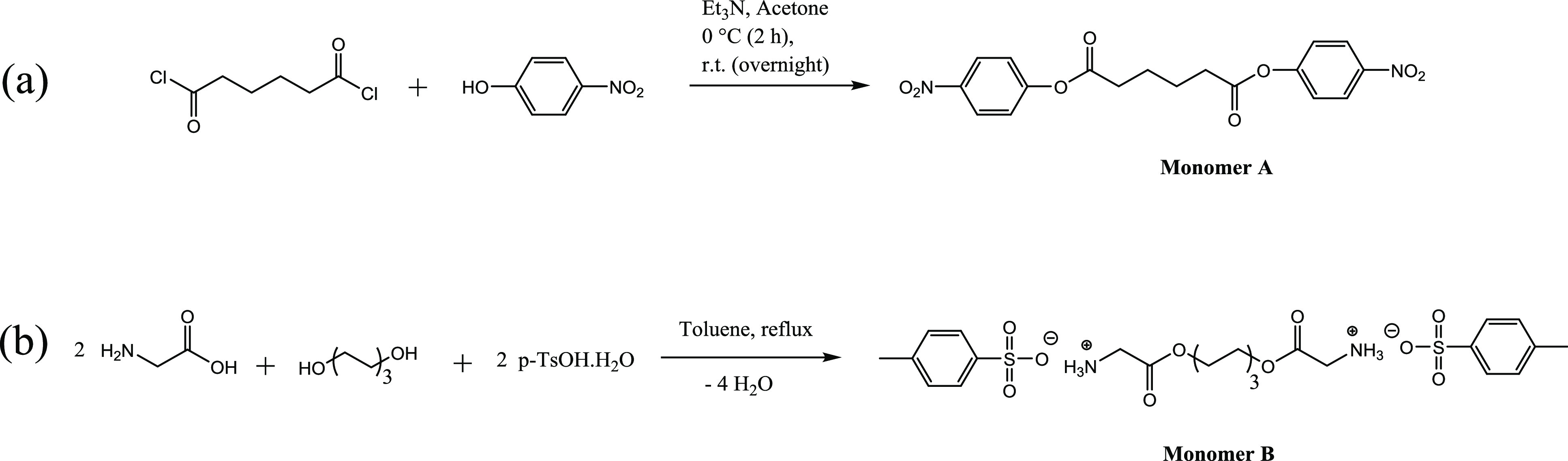
Synthesis Route for (a) Di-*p*-nitrophenyl Adipate (Monomer A), and (b) Di-*p*-toluenesulfonic Acid Salt of Bis(glycine)-hexane 1,6-Diester (Monomer B)

Scheme 1a illustrates the route for the synthesis of monomer A according to the procedure reported by Guo et al.^41^ Briefly, a 2 L three-necked flask, equipped with an overhead PTFE stirring shaft and a CaCl_2_ drying tube, was flushed with dry nitrogen flow for 1 h. Then, a solution of *p*-nitrophenol (452.25 mmol) and dry triethylamine (452.25 mmol) in 1 L of dry acetone was prepared under stirring and the flask was placed in an ice bath. A solution of adipoyl chloride (225 mmol) in 100 mL of dry acetone was prepared and added dropwise by a dropping funnel while the mixture was stirring. After the completion of the addition, the reaction mixture further stirred at around 0 °C for 2 h, and subsequently at room temperature overnight. Finally, the reaction mixture was precipitated in cold distilled water, and the solid product was filtered over a Buchner filter. In the end, the product was washed with 8 L of distilled water, and dried in a vacuum oven at 50 °C overnight. For further purification, repeated recrystallization (7 times) of the product from acetonitrile was performed, and finally, crystals with an off-white color were obtained after drying the product at 50 to 100 °C in the vacuum oven for 24 h. Yield: 87%, DSC: *T*_m_ (peak) = 123.3 °C. ATR-IR ν (cm^–1^): 1750 [(C=O) of the ester bond, stretching], 1200 [(C–O) of the ester bond, stretching], 1526, and 1344 [(NO_2_), stretching]. ^1^H NMR (300 MHz, DMSO-*d*_6_, TMS, int. ref.) δ (ppm): 1.77 (m, 4H, -OCO-CH_2_-CH_2_-), 2.50 (m, DMSO), 2.73 (t, 4H, -OCO-CH_2_-CH_2_-), 3.31 (s, H_2_O), 7.44, 7.47 (4 H, -O-Ph-), 8.29, 8.32 (4 H, -Ph-NO_2_) (Figure S-1). ^13^C NMR (75 MHz, DMSO-*d*_6_, TMS, int. ref.) δ (ppm): 23.40 (-CH_2_-CH_2_-CH_2_-), 33.05 (-CO-CH_2_-CH_2_-), 39.52 (DMSO), 123.13, 125.22 (aromatic carbons), 144.96 (-Ph-NO_2_), 155.35 (-Ph-O-), 171.00 (-CO-) (Figure S-2).

#### Synthesis of Di-*p*-toluenesulfonic Acid Salt of Bis(glycine)-Hexane 1,6-Diester (Monomer B)

Monomer B was synthesized according to the method reported previously^42^ with the application of some changes (Scheme 1b). Glycine (522.72 mmol), 1,6-hexanediol (237.60 mmol), pTsOH·H_2_O (522.72 mmol), and 1200 mL of dry toluene were placed in a 2 L three-necked flask equipped with a nitrogen inlet, a mechanical stirrer, a Dean–Stark apparatus, and a reflux condenser with a CaCl_2_ drying tube on the top. The reaction mixture was heated to reflux for 24 h until around 18 mL of water was collected at the bottom of the Dean–Stark. After that, first the heating and then the stirring were stopped, and the reaction mixture was cooled down slowly. Then, the viscous product, with a light yellowish color settled down at the bottom of the flask, was decanted, and washed twice with diethyl ether while stirring. A whitish solid product was obtained that was dried in the vacuum oven at 40 °C overnight in order to remove the residual solvents. The monomer was dissolved in methanol/water (70/30, v/v) at room temperature until a saturated solution was obtained. Then, the mixture was heated up to 50–60 °C while stirring until a transparent solution was obtained. Subsequently, the heating and stirring stopped and the solution was inspected carefully for complete dissolution of the monomer. The transparent solution was kept at room temperature until recrystallization completed. For further purification, successive recrystallization from methanol/water (70/30, v/v) was done until no impurity peak was observed in the ^1^H NMR spectra. The final product, a white crystalline powder, was dried at 50 to 100 °C gradually in the vacuum oven for 48 h. Yield: 94%, DSC: *T*_m_ (peak) = 120.2 °C. ATR-IR ν (cm^–1^): 1736 [(C=O) of the ester bond, stretching], 1204 [(C–O) of the ester bond, stretching], 2800–3000 [(N–H amine salt, stretching, broad peak)], 1031 and 1118 [(SO_3_), stretching]. ^1^H NMR (300 MHz, DMSO-*d*_6_, TMS, int. ref.) δ (ppm): 1.34 (m, 4H, -(CH_2_)_2_-(CH_2_)_2_-(CH_2_)_2_-), 1.60 (m, 4H, -O-CH_2_-CH_2_-), 2.29 (s, 6H, -Ph-CH_3_), 2.50 (m, DMSO), 3.33 (s, H_2_O), 3.81 (s, 4H, -OCO-CH_2_-NH_3_^+^-), 4.14 (t, 4H, -CH_2_-CH_2_-OCO-), 7.10, 7.13 (4H, -Ph-CH_3_), 7.47, 7.50 (4H, -SO_3_^–^-Ph-), 8.18 (s, 6H, NH_3_^+^-) (Figure S-3). ^13^C NMR (75 MHz, DMSO-*d*_6_, TMS, int. ref.) δ (ppm): 20.79 (CH_3_-Ph-), 24.80 (-(CH_2_)_2_–(CH_2_)_2_-(CH_2_)_2_-), 27.85 (-OCO-CH_2_-CH_2_-), 39.52 (DMSO), 39.76 (OCO-CH_2_-NH_3_^+^-), 65.34 (-CH_2_-CH_2_-OCO-), 125.50, 128.14, 137.92, 145.25 (aromatic carbons), 167.65 (O-CO-CH_2_-) (Figure S-4).

### PEA Synthesis (Screening of *M*_w_ During the Time)

A model polymerization reaction was performed while several samples were taken over the reaction time in order to monitor the changes of the molecular weight during the time. To a three-necked flask equipped with a mechanical stirrer and a nitrogen inlet, the NMR pure and dry monomers of di-*p*-nitrophenyl adipate (5.20 mmol) and di-*p*-toluenesulfonic acid salt of bis(glycine)-hexane 1,6-diester (5.20 mmol), and 8.7 mL of dry DMSO were placed, and the mixture was stirred at room temperature. Then, triethylamine (TEA; 11.44 mmol), freshly dried through refluxing over CaH_2_, was added dropwise (during 1 min) to the sealed reaction flask. The reaction was continued by placing the flask in an oil bath at 60 °C. Several samples were collected from the reaction solution via syringes from the beginning of the polymerization, after the addition of TEA started, until the end of the reaction. The samples that were taken were transferred into a vial and quenched immediately via freezing the vial in liquid nitrogen and then stored at −20 °C.

### Synthesis and End-Capping of PEAs with Different *M*_w_s

**Scheme 2:**
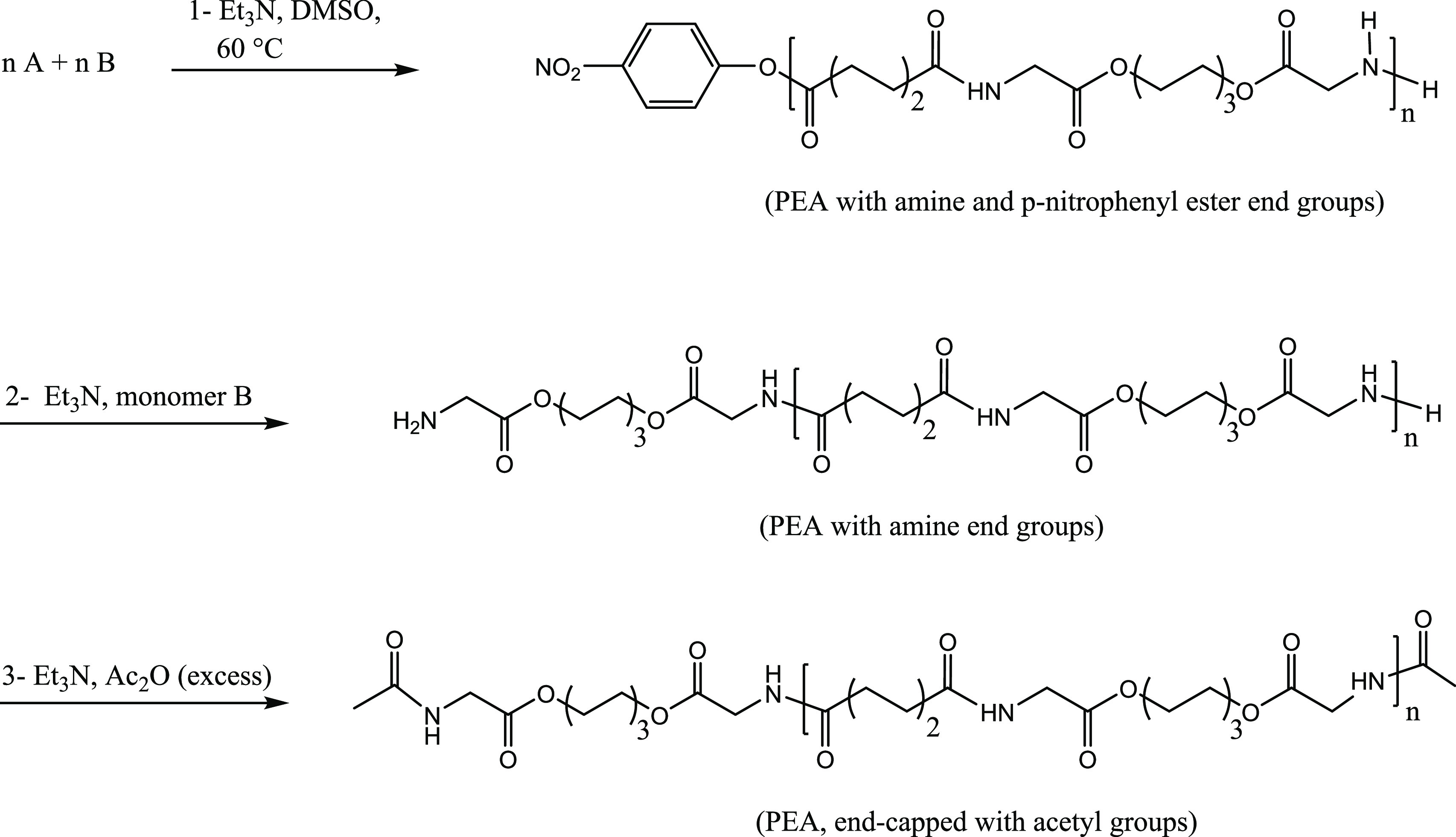
General Procedure for the Synthesis and End-Capping of PEAs

Three PEAs with the same chemical structure and different molecular weights were synthesized via the solution polycondensation of monomers A and B and end-capped with acetic anhydride. According to the model reaction performed previously, each PEA synthesis reaction was stopped after a certain amount of time based on the target molecular weight. Scheme 2 shows the procedure for the synthesis and end-capping of the PEAs. Briefly, dry monomer A (78 mmol) and dry monomer B (78 mmol) together with 130 mL of dry DMSO were added to a 500 mL three-necked flask equipped with an overhead Teflon stirrer shaft and a nitrogen inlet. Then, 171.6 mmol of dry TEA was added dropwise to the sealed flask while stirring at room temperature. The reaction flask was then immersed in an oil bath, thermostated at 60 °C, and the polymerization reaction proceeded for a certain time.

Before starting the end-capping, the reaction mixture was diluted via the addition of 130 mL of dry DMSO, and subsequently, around 10% of the product (nonend-capped PEA) was taken from the flask via a syringe and precipitated in ethyl acetate. In order to end-cap the PEA left in the reaction flask, first, an extra amount of monomer B (6.24, 3.12, and 1.56 mmol, the lower the target molecular weight, the higher the amount of the monomer B added) and the corresponding amount of Et_3_N (mol ratio of Et_3_N/monomer B, 2.2:1) were added to the reaction mixture in order to react with the existing *p*-nitrophenyl ester end groups in the polymer chains for obtaining a PEA with amine end groups. The reaction continued for 2 h, then the PEA was end-capped via the addition of an excess amount of acetic anhydride (30 mL) and TEA (5 mL) and stirring for 1 h at 60 °C and then 2 h at 40 °C. Finally, the end-capped PEA was precipitated in ethyl acetate. The non-end-capped and end-capped PEAs were further purified via dissolving in DMSO and precipitation in ethyl acetate and water, respectively. The purified PEAs were dried in the vacuum oven at 35 °C for 3 days and then at 80 °C for 6 h.

### Materials Characterization

#### Chemical Analysis

^1^H and ^13^C NMR spectra were recorded at ambient probe temperature via a Bruker Avance III-HD Nanobay apparatus at 300 MHz for ^1^H NMR and 75 MHz for ^13^C NMR at ambient probe temperature using DMSO-*d*_6_ as solvent. ^1^H NMR experiments were recorded with 16 scans and ^13^C NMR experiments were recorded with 1024 scans. Chemical shifts are reported in ppm. FT-IR spectra from the powders of the monomers and polymer were collected on a Shimadzu IR Affinity Single Reflection ATR FT-IR spectrophotometer at a resolution of 2 cm^–1^ with 32 scans. The molecular weights of the polymers were measured via gel permeation chromatography (GPC) using 1,1,1,3,3,3-hexafluoroisopropanol (HFIP) with 0.019% NaTFA salt as the eluent (flow rate: 0.33 mL/min) and poly(methyl methacrylate)s (PMMA) as the standards for calibration. Two packed PFG combination medium columns (particle size: 7 μm, separation range: 100–1000000 Da) with a PFG precolumn (particle size: 7 μm) were utilized, and the polymers were detected using a SECcurity refractive index (RI) detector. In order to prepare samples for the injection (injection volume: 10 μL), polymers were dissolved in HFIP with 0.019% NaTFA at room temperature (about 3 mg/mL), and subsequently were filtered over a 0.2 μm PTFE syringe filter into a GPC vial. The molecular weight and dispersity (*Đ*) values of the polymers were calculated with respect to the PMMA standards through the evaluation of the elution diagrams via PSS WinGPC software from Polymer Standards Service (PSS).

#### Thermal Analysis

The thermogravimetric analysis (TGA) of the samples was performed with a TA Instruments Q500 thermogravimetric analyzer under a nitrogen atmosphere using a heating rate of 10 °C/min from 25 to 700 °C. The polymers were also analyzed via differential scanning calorimetry (DSC) using a Netzsch Polyma 2014 DSC calibrated with indium, tin, bismuth, and zinc. The samples were subjected to heating/cooling for two cycles with a heating/cooling rate of 10 °C/min under a nitrogen flow of 20 mL/min. The *T*_g_ was determined by measuring the midpoint temperature. The melting point of the monomers was measured from their first heating cycle with a heating rate of 5 °C/min under nitrogen flow.

### Rheology

The melt rheology of the polymers was studied via an Anton Paar MCR 702 TwinDrive apparatus with parallel plates (diameter: 25 mm, the gap between the plates: 500 μm) under a nitrogen flow. In order to decrease the loading time of the polymers between the plates and to apply the same thermal history, tablets of the PEAs were used instead of their powders. PEA tablets with a diameter of 13 mm were made by placing the PEA powder into an Evacuable Pellet Die (Specac, Britain) and pressing them under a load of 10 tons via a Specac manual hydraulic press for 1 min at room temperature. The tablets were dried in a vacuum oven at 80 °C for 6 h before use.

Melt complex viscosity as a function of temperature, an important parameter in 3D printing, was measured by applying an oscillatory strain up to 1%, at a frequency of 1 rad/s, while continuously decreasing the temperature at a rate of 5 °C/min. The ramp was started from 200 °C and stopped when the complex modulus exceeded 10^6^ Pa due to crystallization, preventing machine damages. The thermal stability of the melts was evaluated from the trend of complex viscosity over time upon the application of a series of frequency sweeps for 2 h at a constant temperature (200 °C). The maximum strain was set at 1% and the frequency was varied between 100 and 5 rad/s in each sweep. The data were extrapolated in terms of the value of viscosity at a specific frequency over time.

### Scaffold Fabrication

AM of the PEAs was performed via a Bioscaffolder (SysENG, Germany) with a G22 nozzle (inner diameter: 413 μm). First, the CAD model of the samples to be printed was drawn in the 3D computer graphics software Rhinoceros (Robert McNeel and Associates, U.S.A.). This was a cylinder with diameter and height of 8 mm. The numerical control (NC) code for the printer was then generated by slicing the CAD model with the slicing software PrimCAM (Primus Data, Switzerland). The geometrical parameters used were strand distance (*d*_2_) = 0.826 mm, layer thickness (*d*_3_) = 0.33 mm, angle between the layers = 90°, and with no meander. By evaluating scaffolds printed under a specific set of parameters with an optical microscope, pressure applied to the melt, screw RPM, print head translational speed, and extrusion temperature were optimized for each polymeric composition. Once the numerical control (NC) code had been generated, this was imported in the machine-operating software, DiSoft (Axiss, Germany). PEA powder was then loaded into the print head reservoir, allowed to melt, and eventually extruded in the layer-by-layer pattern, as described by the code.

### Scaffold Characterization

The overall morphology of the AM cylinders was analyzed via a Nikon SMZ25 stereomicroscope, while the internal structure was characterized with a Philips XL-30 Scanning Electron Microscope (SEM). To observe the cross section view of the scaffolds, they were cut using a surgical blade and then sputter-coated with a thin layer of gold with a Cressington sputter coater. The theoretical porosity of the scaffolds was calculated according to the previously reported method using eq 1.^43,44^
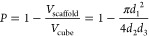
1where *P* is the porosity of the scaffold, *V* is the volume, and *d*_1_, *d*_2_, and *d*_3_ are the fiber diameter, fiber spacing, and the layer thickness, respectively. The measured values for *d*_1_, *d*_2_, and *d*_3_ were obtained from the SEM images of the scaffolds using ImageJ software. The porosity was also measured experimentally via eq 2.^44^
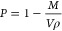
2where *M*, *V*, and ρ are the mass, volume, and the density of the polymer used for 3D printing, respectively. The density of the polymers was measured according to the Archimedes principle using an analytical Mettler-Toledo XSE105 dual range balance with an XPR/XSR-Ana density kit. The weight of the samples was measured first in air and then in ethanol as an auxiliary solvent with a known density. The density of the polymers was calculated via the integrated application on the analytical balance using the following formula:
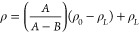
3where ρ is the density of the polymer, *A* is the weight of the polymer measured in air, *B* is the weight of the polymer measured inside ethanol, ρ_0_ is the density of the ethanol at the temperature in which the experiments were performed, and ρ_*L*_ is the density of air.

### Mechanical Tests

In order to measure the tensile properties of the polymers, PEA films were prepared via the compression molding method. The polymer powder was loaded in between two flat metal plates covered by PTFE sheets and a 240 μm thick spacer. Then, the polymers were processed with a “Collin P 200 E” hot press at 30 °C above their melting point for 3 min (under a pressure of 5 bar for 2 min and 40 bar for 1 min). The samples were cooled down to room temperature using a water cooling system. The prepared films were punched into dog-bone-shaped specimens using a cutting device (ISO 527-2 1BB) and a manual punching machine.

The compression mechanical tests of the cylindrical-shaped samples in 3D printed and bulk forms were also studied. The bulk cylinders were made via the compression molding technique using a QS17-102 stainless steel mold with a diameter of 8.1 mm and a height of 12.6 mm made by IDEE (Instrument Development, Engineering and Evaluation) at Maastricht University (Figure S-5-E). In order to prepare the cylindrical-shaped samples, each polymer in powder or granule form was loaded into the mold chamber, and the polymers were isothermally heated to 30 °C above their melting point under a pressure of 5 bar for 3 min. The temperature was then decreased to the melting point of the samples and stayed at that temperature for 20 s. Then, the samples were cooled down to room temperature using the water circulatory cooling system of the device. The typical dog bone, 3D printed cylindrical scaffold, and bulk cylindrical specimen used for the mechanical tests are shown in Figure S-5-A, B, and C, respectively.

The tensile strength tests of the polymer films and the compression tests of the polymer bulk cylinders and cylindrical AM scaffolds were performed on a Zwick/Roell Z020 universal testing machine (Zwick GmbH, Ulm, Germany). For the tensile test, the dog-bone-shaped films with a length of 3 cm and 2 mm width of the center of the dog bone were tested using a load cell with a nominal force of 100 N. While the starting speed for determination of the tensile modulus was set to 1 mm/min, the speed of the remainder protocol was 5 mm/min. The thickness of the films and the height and diameter of the cylindrical samples were measured via a micrometer and the values were manually entered in the measuring software (TestXpert II) for the automatic calculations of the measurements. For the compression test, the cylinders with an approximate diameter of 8 mm were loaded in compression fitted with a 20 kN load cell with a constant true strain speed of 0.001 (1/s). Before running the test, for both of the 3D printed and bulk cylinders, the top and bottom faces of the cylinders were carefully polished to give a flat surface. In order to minimize the friction between the surfaces of the samples and the mechanical tester plates required for a satisfactory deformation of the specimen along the strain range, a layer of PTFE film was placed on the top and at the bottom of the cylinders and a droplet of water-soap mixture as surfactant was applied on the top on bottom of PTFE films, as shown in Figure S-5-D.^45^ True stress was calculated in the assumption of incompressibility.

### In Vitro Evaluation

#### Cell Culture

The interaction of the PEAs and PLGA with the cells was evaluated using human osteosarcoma cell lines (MG-63) as model cells. The cultured cells were maintained in an incubator with humidified atmosphere and 5% CO_2_ at 37 °C using Dulbecco’s Modified Eagle Medium (DMEM, Gibco, U.S.A.) with high glucose concentration and supplemented with 10% (v/v) fetal bovine serum (FBS) as culture medium.

#### Cell Attachment, Viability, and Proliferation

A live–dead assay was performed in order to determine the cell attachment, viability, and proliferation on the polymer films using tissue culture plates (TCP) as positive control. The polymer films made of PEAs and PLGA were cut into disk shapes with a diameter of 10 mm via a manual puncher. The samples were disinfected via soaking in ethanol 70% and then washing with phosphate buffered saline (PBS) twice. A duplicate of the PEAs and PLGA films were placed in three separated well plates with 48 wells (for three time points, days 1, 3, and 7) and all of the samples and the positive controls were maintained in the culture medium for 4 h prior to the cell seeding. Then, the culture medium was removed and the surface of the samples was washed with PBS twice. MG-63 cells were seeded at a density of 10k cells/mL (5k cells/well) and incubated for days 1, 3, and 7. The culture medium was refreshed at days 1, 3 and 5. At the end of each time point, after removing the culture medium and washing the samples with PBS, the samples were stained with Calcein-AM fluorescein and ethidium homodimer-1 (Eth-D1) for the live–dead test following the manufacturer’s instruction. After 30 min of incubation at 37 °C in the dark, the supernatants were removed, the samples were washed with PBS, and the culture medium (without FBS) was refreshed. The samples were transferred to the fluorescent microscope quickly and the images were acquired via a slide-scanner fluorescence microscope.

### Metabolic Activity

The disk-shape polymer samples with a diameter of 10 mm were subjected to cell seeding in 48-well plates. MG-63 cells with a density of 5k cells/well were seeded on the triplicates of samples for each time point. PrestoBlue cell viability reagent (Thermo Fisher Scientific) as a nondestructive substrate was used to quantitatively analyze the proliferation of the cells after 1, 3, and 7 days using DMEM supplemented with 10% FBS as culture medium. The PrestoBlue reagent is reduced into a highly fluorescent compound in contact with the reducing environment of the living cells. The fluorescence measurements of this chemical change indirectly measure the amount of cells in each well. Briefly, the medium was removed from the wells, including the samples and controls, which were then washed with PBS twice. A 10% solution of PrestoBlue diluted in the culture medium was added to each well, and the well plates were incubated at 37 °C for 20 min while covered from light via an aluminum foil. Afterward, the fluorescence of the medium was measured at 590 nm via a PerkinElmer Victor 3 1420 multilabel plate reader. The samples were washed with PBS twice and were kept dry at −80 °C for the DNA measurements.

### DNA Quantification

The quantification of the DNA amount was performed using a CyQuant cell proliferation assay (Life Technologies). The dry samples (*n* = 3) were frozen at −80 °C after the metabolic activity test and then subjected to freeze–thawing three times (30 min in −80 °C and 30 min at room temperature). Next, a lysis buffer (KH_2_PO_4_ (0.1 M), K_2_HPO_4_ (0.1 M), Triton X-100 (0.1%), pH (adjusted to 7.8), and cell scraper were used to release all the cells from the wells. The lysed cells were used for DNA quantification. The lysed samples were transferred to eppendorf tubes and processed for digestion at 56 °C overnight in a 1 mg/mL solution of proteinase K/(Tris/EDTA) buffer. The proteinase K digested samples were frozen using liquid nitrogen and thawed at 56 °C for three times, and then a CyQuant lysis buffer (1×) containing RNase A (1:500 diluted) was mixed 1:1 to each of the samples and left for 1 h at room temperature to degrade the cellular RNA. The quantification of the amount of DNA per sample was performed based on the protocol provided by the CyQuant DNA assay manufacturer (Life Technologies). The fluorescence of the samples was measured via a PerkinElmer Victor 3 1420 multilabel plate reader at emission wavelength of 520 nm and excitation wavelength of 480 nm.

### LDH Cytotoxicity Assay

The cytotoxicity of PEA-MM_w_, PEA-HM_w_, the biomedical grade PLGA, and TCP as control was measured via a CyQUANT LDH cytotoxicity assay kit (ThermoFisher Scientific). Lactate dehydrogenase (LDH) is a cytosolic enzyme that is present inside the living cells, which releases into the culture media after the plasma membrane damage. Measuring the level of the released LDH from the cells seeded on the polymer samples is an indication of the polymer’s cytotoxicity. A series of 48 well plates were used for the cell seeding on the polymer discs with a diameter of 10 mm using high glucose DMEM with 10% FBS as culture media. For each time point, next to the triplicates of polymer samples, triplicates of cell-seeded TCPs for measuring the spontaneous LDH release (negative control) and an additional triplicate of cell-seeded TCPs for measuring maximum LDH activity control (positive control) were included. The plates were placed in an incubator with a humidified atmosphere and 5% CO_2_ at 37 °C. The supernatants were collected at the end of days 1, 3, 5, and 7 and then refreshed. Before collecting the supernatant, in order to determine the 100% LDH activity needed for the final calculations, 20 μL of lysis buffer (10×) from the LDH cytotoxicity kit was added to the set of triplicate wells serving as the maximum LDH activity. A total of 20 μL of Milli-Q water was added to the polymer-treated samples and the negative control samples, and then the well plates were placed in the incubator for 45 min. All of the collected media at the end of each time point were kept at −80 °C for the cytotoxicity measurements according to the protocol provided by the kit producer. For the final measurements, 8 μL of each sample was transferred to flat bottom 96-well plates and was diluted to 50 μL via the addition of PBS. Then, 50 μL of the reaction mixture, prepared according to the kit protocol, was added to each sample. The plates were protected from light and incubated at room temperature for 30 min. Subsequently, 50 μL of the stop solution from the kit was added to each sample, and the absorbance of the samples was measured at 490 and 680 nm. The absorbance values at 680 nm (background) were subtracted from the 490 nm values before performing the calculations based on the formula below:
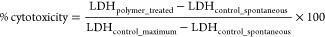


### Bioactivity Evaluation Using SBF

The SBF was prepared according to the procedure described by Bohner et al., which was to simplify the composition of the SBF and mimic the main features of the blood serum by using only the essential inorganic ions which exist in the blood serum in our SBF.^46^ In order to avoid the formation of premature hydroxyapatite (HA) or any other precipitations during the preparation and storing of SBF solution, the new method suggested by Bohner et al. was applied. The SBF composition was divided into two separated flasks named solution A and B. The composition of solution A and B is shown in Table S-1. The solutions A and B were made under sterile conditions and filtered over a 0.2 μm sterile filter into sterile plastic flasks and stored at 4 °C. The pH of the SBF after mixing an equal amount of solutions A and B needs to be close to 7.4 at 37 °C, otherwise the pH of the solution A or B in the main flasks needs to be adjusted gradually by HCl (1.0 M) in order to obtain the target pH accordingly. In order to do the bioactivity evaluation of the samples, the polymer discs with a diameter of 14 mm and heights of about 210–230 μm, which were manually punched out of the PEA-MM_w_, PEA-HM_w_ and PLGA films, were first disinfected via soaking in ethanol (70%) for 20 min, then washed with PBS twice and then transferred into 6 well plates. Duplicates of samples were used for different time points of 4, 7, 14, and 21 days. In order to expose the samples to the SBF, an equal amount of the solution A and B (2 mL) was added simultaneously to the samples and then the well plates were maintained in an incubator at 37 °C with 5% CO_2_ for a certain time. The SBF solution was removed and refreshed every 48 h during the experiment. At the end of each time point, the samples were collected, gently washed with pure water, and then dried at ambient conditions for several days. The formation of hydroxyapatite on the surface of the samples and their morphology was studied using a Philips XL-30 Scanning Electron Microscope (SEM). The samples were gold sputtered under a current of 40 mA/mbar for 70 s. The composition of the inorganic compound mineralized on the surface of the films was analyzed by powder X-ray diffraction (XRD) using a Bruker D2 Phaser (Cu Kα radiation, λ = 0.15405 nm). For a better understanding of the mineralization of hydroxyapatite on the surface of PEAs, the formation and morphology of the hydroxyapatite on the surface of PEA-MM_w_ and PEA-HM_w_ in 3D printed form was also studied.

## Results and Discussion

### 1 Poly(ester amide) synthesis

**Figure 1:**
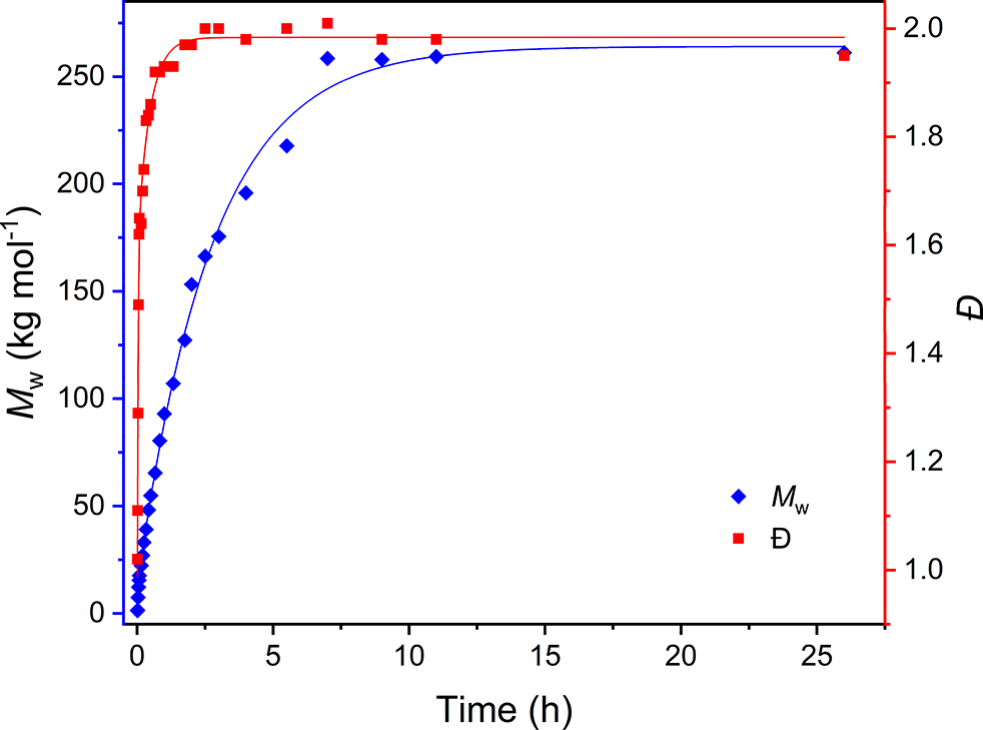
Evolution of molecular weight (*M*_w_) and dispersity (*Đ*) as a function of time for PEAs made via active solution polycondensation with a 1:1 feed ratio of monomer A/monomer B. The exponential fit of *M*_w_ (ExpGro2, R-Square = 0.996) and *Đ* (ExpGro2, R-Square = 0.986) were extrapolated via Origin software.

In order to study the effect of the molecular weight of the PEAs on FDM 3D printing, three different molecular weights were synthesized. The PEAs were synthesized via the active solution polycondensation of the monomers A and B in a mole ratio of 1:1 under dry conditions. To achieve any of the target molecular weights, the reactions were stopped at different time points based on the curves shown in Figure 1. In order to study the evolution of the molecular weight and dispersity of PEA during the reaction time, samples were withdrawn at regular time intervals and their molecular weight and dispersity were measured via GPC. As illustrated in Figure 1, the polycondensation procedure went from oligomers to plateau values for the molecular weight within approximately 15 h. The dispersity values during the reaction gradually evolved toward 2, typical values for polycondensates.

The molecular parameters of the purified PEAs with different molecular weights are listed together with a commercial PLGA (PLG 8218) in Table 1. PLG 8218, kindly provided by Corbion Purac Biomaterials, is a semicrystalline PLGA with a lactic acid/glycolic acid (LA/GA) mol ratio of 82:18 and serves in this study as comparative polymer as typically PLGA samples are among the frequently used biocompatible polymer resources in the fabrication of fused deposition modeled scaffolds. PEA-LM_w_, PEA-MM_w_, and PEA-HM_w_ listed in Table 1 stand for the PEAs with low, medium, and high *M*_w_ values, respectively.

**Table 1:** Molecular Parameters of the PEAs Synthesized with an Equimolar Feed Ratio of Monomers A and B and the Commercial PLGA as the Comparative Polymer

sample name	reaction time (min)	remark	*M*_w_^a^ (g/mol)	*Đ*	mol % Ain polymer^b^	mol %B in polymer^b^
PEA-LM_w_	10	non-end-capped	21800	1.9	50.0	50.0
		end-capped	24700	1.9		
PEA-MM_w_	50	non-end-capped	52900	2.0	49.9	50.1
		end-capped	60100	2.0		
PEA-HM_w_	90	non-end-capped	101500	2.4	49.9	50.1
		end-capped	122400	2.4		
PLGA			209300	2.3		

a GPC in HFIP/0.19% NaTFA, RI detection.

b The mol % of each monomer in the product was calculated using MestReNova software based to the calculated integrals of the peaks e (for monomer B) and d (for monomer A) in the ^1^H NMR spectra of the polymers (Figure 2) at different molecular weights.

As during FDM, where the polymers are subjected to temperatures above their melting point for a relatively long time, they are susceptible to chemical reactions during processing. The presence of free amine and *p*-nitrophenyl ester end groups in the synthesized PEAs can lead to further polymerization and transreactions within the polymer chains, especially at high temperatures. In order to reduce these possible chemical reactions, the PEA chain ends were deactivated by end-capping. Therefore, at the end of the reaction, amine-functional PEA was made by the addition of excess monomer B as well as TEA, after which those reactive amine groups were end-capped via acetylation with acetic anhydride. ^1^H NMR analysis of PEA-LM_w_ before and after end-capping (Figure 2) confirmed chemically the successful end-capping with acetic anhydride, as visible from the appearance of a small singlet peak at 2.07 ppm due to the CH_3_ group of the acetyl end groups in end-capped PEA. As the ratio of the repeating units to the acetyl end groups increased with the increase of the molecular weight, the mentioned peak for the protons of the CH_3_ of the acetyl group did not appear for the end-capped PEAs with higher molecular weights. The small singlet at 1.99 ppm belongs to ethyl acetate residuals in the polymer. The ^1^H NMR spectra were inspected for the presence of triethylamine in the product, and no indication of characteristic peaks for triethylamine in DMSO-*d*_6_ (0.93 and 2.43 ppm) was found. In order to evaluate the possible presence of DMSO in the product after the purification procedure, a ^1^H NMR spectrum in DMF-*d*_7_ was recorded. According to the results (Figure S-6), a small singlet peak appeared at 2.59 ppm, which was attributed to DMSO residuals. The calculated amount of DMSO in the product was about 0.6 wt %, as shown in Figure S-6. Considering at least 10× further dilution of the DSMO during the cell culture procedure, the cytotoxic effect of the DMSO traces on the MG63 cells is expected to be negligible. The ^13^C NMR spectra of the synthesized PEA, shown in Figure S-7, further confirmed the chemical structure of the synthesized PEA. However, due to the low number of the acetyl end groups and low sensitivity of the ^13^C NMR, the peak of the carbon of the acetyl group in the end-capped PEAs was not observed in the spectra.

**Figure 2:**
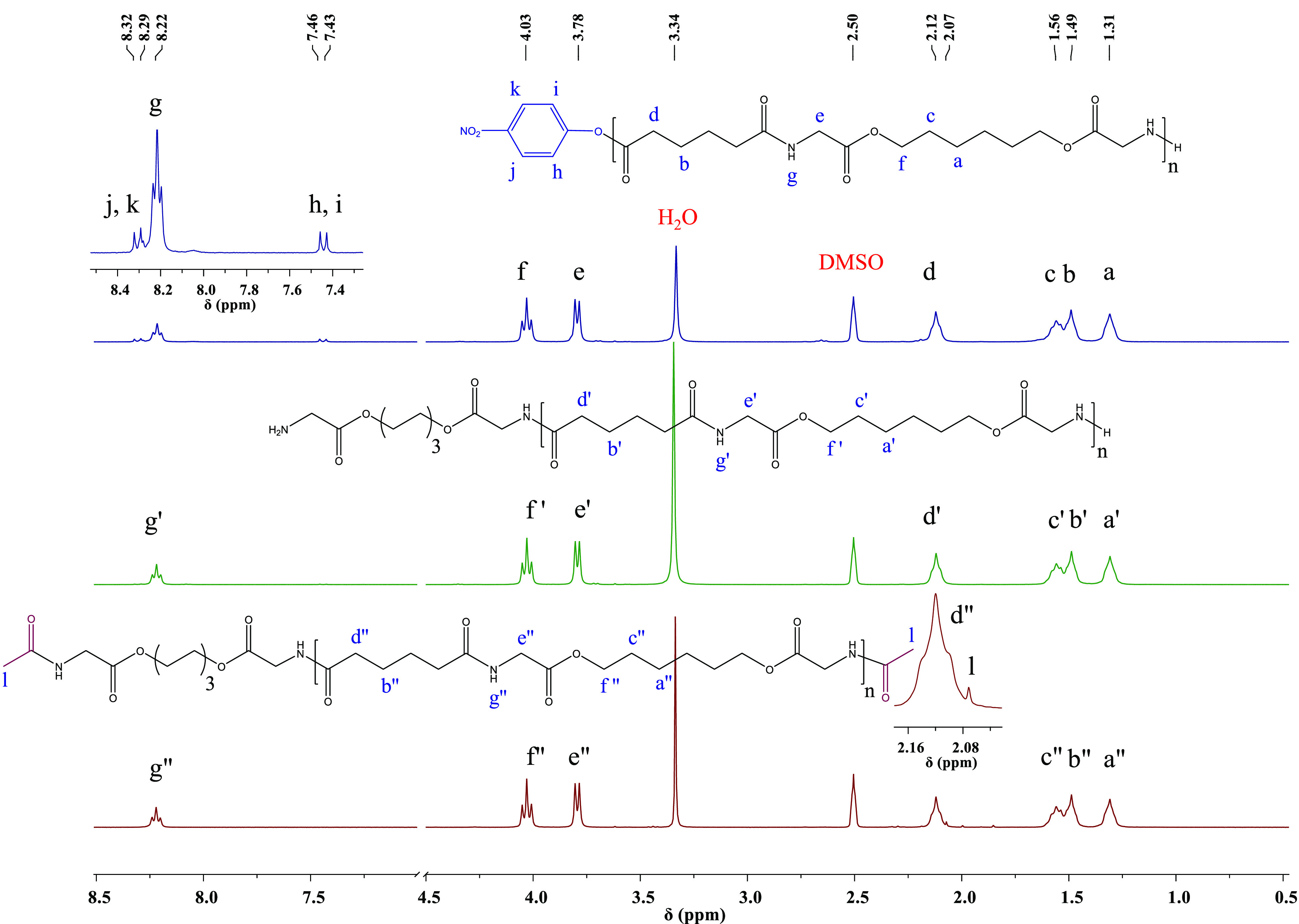
^1^H NMR spectra of the PEA-LM_w_, before end-capping (top), after pre-end-capping with monomer B (middle), and after end-capping (bottom).

### 2 Thermal Characterization by TGA and DSC

In order to compare the thermal stability of the PEAs with PLGA, TGA curves were recorded for the end-capped PEAs. As shown in Figure S-8 and Table 2, the PEAs exhibited higher stability against thermal degradation compared with PLGA. This can be attributed to the presence of amide bonds in the PEA structure, which are known to have higher stability than ester bonds.^27^ In addition, the presence of the amide bond increases the intermolecular hydrogen bonding between the (-NHCO-) groups improving the thermal stability of PEAs compared with polyesters.^47^ The temperature at which 5% weight loss occurred (*T*_5%_) for the samples are shown in Table 2. The T_5%_ for PLGA is about 323 °C while the T_5%_ for PEA-LM_w_ to PEA-HM_w_ varies from about 349 to 361 °C.

**Table 2:** Thermal Properties of the Synthesized PEAs and PLGA

sample	remark	*T*_g_ (mid; °C)	*T*_m_ (peak; °C)	*T*_c_ (peak; °C)	Δ*H*_m_^a^ (J/g)	Δ*H*_c_ (J/g)	*T*_5%_ (°C)
PEA-LM_w_	end-capped	18	164	130	86.6	82.7	349
PEA-MM_w_	end-capped	20	167	129	77.3	73.9	357
PEA-HM_w_	end-capped	20	165	125	61.0	58.8	361
PLGA^b^		57	141		40.4		323

a The net enthalpic effect, not discriminating the reorganization processes and final melting.

b The thermal properties of PEAs was determined from their second heating/cooling cycle, while for PLGA, the reported values are based on its first cycle, as it did not crystallize upon first cooling.

**Figure 3:**
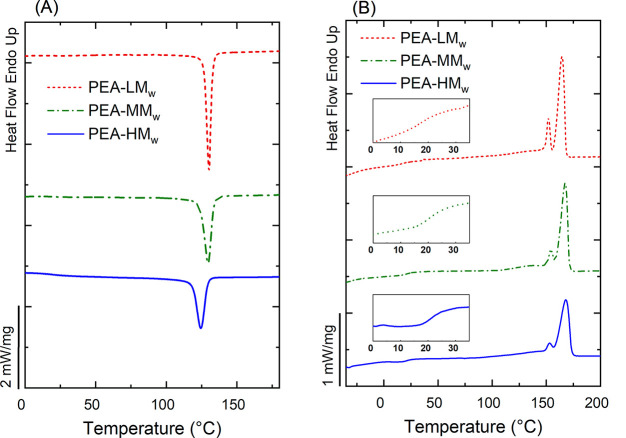
DSC thermograms of the PEAs with different molecular weights, second cooling (A) and second heating (B) run at 10 °C/min rate for the indicated samples.

According to the DSC thermograms (Figure 3), the PEAs possess a semicrystalline behavior with a glass transition temperature (*T*_g_) as well as a sharp melting (*T*_m_) upon heating and crystallization temperature (*T*_c_) once successively cooled. The small endothermic event observed prior to the melting peak in all of the PEAs was attributed to the reorganization of the crystals before melting, as confirmed by recording the heat flow for the PEAs with higher cooling rates (Figure S-9). Observation of cold crystallization prior to melting in the second heating (10 °C/min), after being cooled with a 30 °C/min rate in the first cooling from the melt, supported the reorganization of the crystals prior to melting.^48,49^ The DSC thermograms of PLGA (Figure S-10), which is a semicrystalline polymer as well, showed a *T*_g_ and melting peak in the first heating cycle. However, the polymer did not crystallize under a 10 °C/min cooling rate. Therefore, the polymer showed an amorphous behavior in the second cycle. This behavior could have an effect on the physical and mechanical properties of the additively manufactured scaffolds as crystallization may not occur under the cooling rates imposed by FDM. In this perspective, the high crystallization rate of the PEAs can be considered as an advantage.

As shown in Table 2, the *T*_g_, *T*_m_, and *T*_c_ of the semicrystalline PEA with different molecular weights were relatively close to each other. The minor changes in the *T*_g_ of the PEAs with a considerable difference in molecular weight shows that, within the synthesized range, the molecular weight does not affect thermally induced segmental motion in the amorphous phase. The enthalpies of melting and crystallization of the PEAs indicate that under these conditions the crystallinity decreases with increased molecular weights. Since the *T*_m_ of the polymers plays a key role in AM via the FDM method, it is important to synthesize the PEAs with relatively low *T*_m_ in order to minimize their thermal degradation, especially in the ester bond sites, during the printing time. The synthesized PEAs with a relatively low *T*_m_ between 164 and 167 °C were expected to be suitable for extrusion-based AM. As the thermal properties of the PEAs are related to the nature of the amino acid and the length of the aliphatic chain in the structure of the diol and di-p-nitrophenyl ester used as the starting materials,^32^ the PEAs chemical structure could be designed in different ways in order to tune their crystallinity and thermal properties. These variations can change the mechanical properties accordingly. This tunability can be used as an advantage in the design and utilization of PEAs with different physical properties for AM.

### 3 Rheology

**Figure 4:**
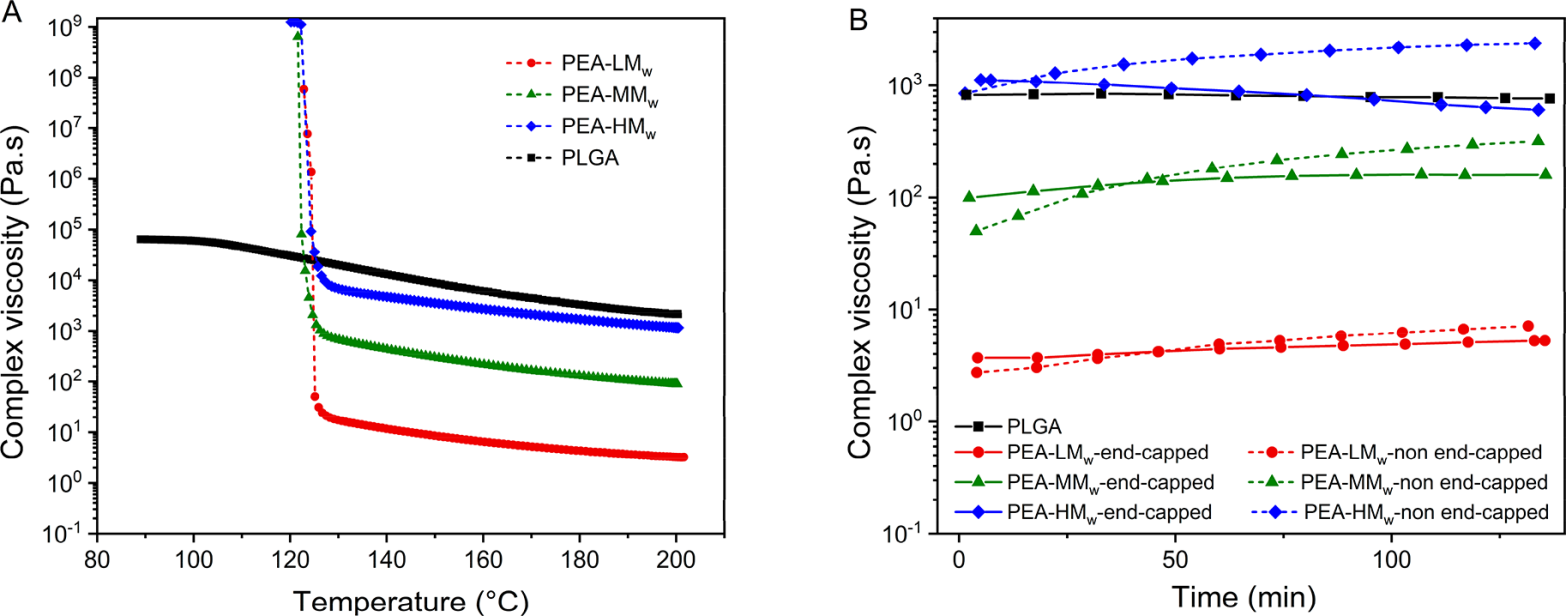
Complex viscosity changes of the end-capped PEAs and PLGA upon a dynamic cooling ramp (A) and variations of the complex viscosity of the non-end-capped PEAs, end-capped PEAs, and PLGA during the time at 200 °C, angular frequency of 100 rad/s, and shear strain of 1% (B).

The rheological behavior of the PEAs was studied in comparison to a commonly used biomedical grade PLGA. The samples were cooled from the melt in order to study the influence of temperature on complex viscosity. The obtained data provide insight on how to design the AM process. Under the applied cooling rate of 5 °C/min, the distinct increase in complex viscosity in the range of 120 to 130 °C was caused by crystallization of the PEAs while PLGA did not crystallize in the studied temperature range (Figure 4-A). As expected, the difference in the molecular weight of the PEAs clearly affected their complex viscosity values, which is an important factor for their AM procedure as it influences extrudability, interfacial bonding, solidification and shape retention.^50^ However, considering (i) the cooling rate in the rheometer to be distinctly lower than during melt deposition in FDM and (ii) heat transfer of successively added filaments, and so cold crystallization upon reheating is minimized,^51^ PLGA likely remained amorphous in the final scaffold. Controlling crystallization and final crystallinity are, next to the glass transition temperature, important parameters in designing the mechanical properties of polymeric products^52,53^ and so of scaffolds.

Another key factor for the AM of polymers with the current technique is their thermal stability during deposition. As the material is molten in the reservoir and then extruded, the stability over time of its rheological properties is essential to have a uniform and reproducible process. In order to evaluate the thermal stability, series of isothermal frequency sweeps (200 °C) were performed on the end-capped and non-end-capped PEAs, and possible changes of complex viscosity over time were measured. As shown in Figure 4B, the complex viscosity changes showed two different trends for the end-capped and non-end-capped PEAs. The complex viscosity of the non-end-capped PEAs increased as a function of time at the isothermal temperature of 200 °C, which could be due to the further melt condensation of the free amine and *p*-nitrophenyl ester functional groups present in the polymer melt mixture. Initially, the complex viscosity of the non-end-capped samples was somewhat lower than the corresponding end-capped ones. This is likely due to the moderately higher molecular weight of the end-capped PEAs, as previously shown in Table 1.

The increase in complex viscosity of the non-end-capped PEAs over time may be a result of linear chain growth, cross-linking, or a combination of both. The GPC results after the stability test (Table S-2) also confirmed that the molecular weight and dispersity of the non-end-capped samples increased significantly compared with the end-capped ones. Furthermore, post analysis of the rheometry samples showed that the non-end-capped samples of PEA-HM_w_ did not completely dissolve in GPC solvents and swelling of the sample was observed instead. This indicates the cross-linking of PEA-HM_w_, which limits melt processing and in particular fused deposition modeling from a pressurized melt reservoir. On the other hand, the complex viscosity of the end-capped PEAs showed higher stability over the time-scale of the test. The GPC results after the test confirmed that their molecular weight did not change significantly in comparison with the non-end-capped samples. This makes the end-capped samples more suitable for melt-based 3D printing. The complex viscosity of the commercial PLGA sample also remained relatively stable during the test. Although the molecular weight of the end-capped PEA-HM_w_ was much lower than PLGA, their complex viscosities were in a similar range, which could be due to the higher hydrogen bonding between the PEA chains compared with PLGA.

**Figure 5:**
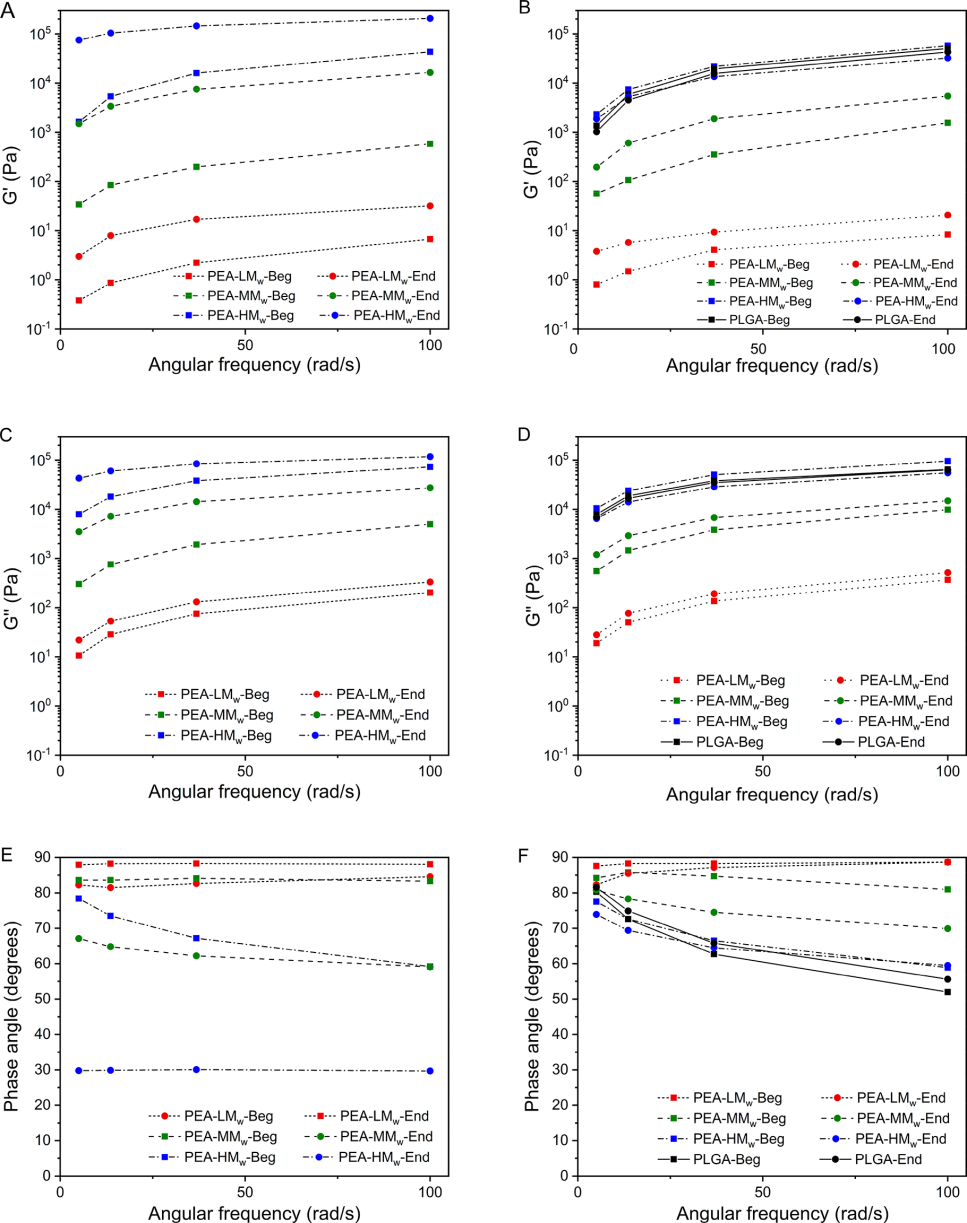
Comparison of the storage modulus (*G*′), loss modulus (*G*″), and phase angle in the beginning (Beg) and end (End) of the test for the non-end-capped PEAs (left, A, C, and E) vs the end-capped PEAs and PLGA (right, B, D, and F). Frequency sweep (5–100 rad/s) at 200 °C, (test time ∼ 2.5 h). The loading time of the samples including the sample’s trimming and temperature stabilization before the beginning of the measurements was 10–15 min.

The changes of the storage modulus (*G*′) and loss modulus (*G*″) of the PEAs at the beginning and the end of the stability test at 200 °C, over the frequency sweeps, were also studied for a better understanding of their viscoelastic behavior in the end-capped and non-end-capped states. As illustrated in Figure 5A, the *G*′ of the non-end-capped PEAs at the end (End) of the test was increased significantly in comparison with the beginning (Beg) of the test, implying that the elasticity of the samples was increased as a result of cross-linking. On the other hand, the changes in *G*′ for the end-capped samples were considerably less than the corresponding nonend-capped ones (Figure 5B). As the complex viscosity of the non-end-capped polymers increased, as a result of melt-condensation and cross-linking, their *G*″ values also increased, which indicates an increase in the viscous behavior as time proceeds. This increment for the end-capped samples is much less because of less condensation reactions in the end-capped PEAs during the stability test (Figure 5C vs D). The commercial PLGA also showed very small changes in the viscoelastic behavior. The changes in the phase angle from the beginning to the end of the stability test (Figure 5E,F) further revealed the differences in the viscoelastic behavior for the non-end-capped PEAs over time in comparison with the end-capped ones and PLGA. The phase angle of the non-end-capped PEAs with higher *M*_w_ decreased further toward the end of the test due to cross-linking between their longer chains with higher entanglement density. In contrast, the relatively small changes in the phase angle values for the end-capped PEAs and PLGA further showed the higher stability of the viscoelastic behavior of those samples during the experimental time. According to these results, not only the end-capping of the PEAs improves the steadiness of the viscoelastic behavior, but it also seems to be necessary for keeping the AM quality of the polymers consistent during the printing time.

### 4 Scaffold Fabrication via AM

**Figure 6:**
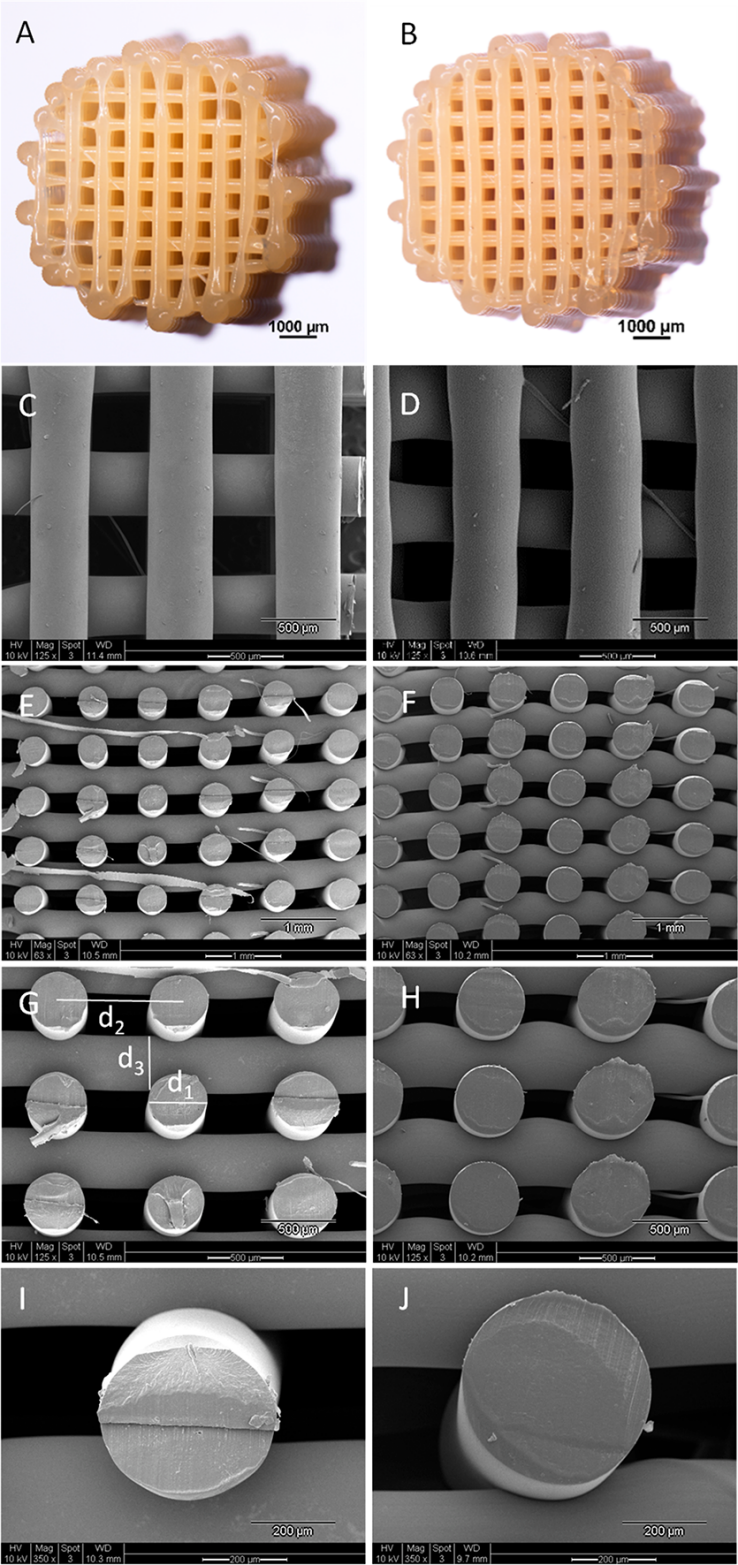
Stereo microscopic images of the 3D printed scaffolds of PEA-MM_w_ (A) and PEA-HM_w_ (B); SEM micrographs of PEA-MM_w_ (top view (C) and cross section (E, G, and I) at different magnifications) and PEA-HM_w_ (top view (D) and cross section (F, H, and J) at different magnifications). The fiber diameter, fiber distance, and layer thickness are shown as *d*_1_, *d*_2_, and *d*_3_, respectively (G).

Cylindrically shaped 3D scaffolds of the end-capped PEA-MM_w_ and PEA-HM_w_ were fabricated using a Bioscaffolder AM device. Due to the low complex viscosity of PEA-LM_w_, dimensional control of printed scaffolds was challenged and not feasible. A summary of the parameters used for 3D printing of PEA-MM_w_ and PEA-HM_w_ can be found in Table S-3. The molecular weights of the PEAs after 3D printing did not change significantly as proven by the GPC results of the PEAs scaffolds shown in Table S-2. Representative stereo microscopic photos and SEM micrographs of the 3D printed PEA-MM_w_ and PEA-HM_w_ are shown in Figure 6. The SEM images of both PEA scaffolds show filaments without any cracks or pores with a homogeneity in layer thickness and shape for both. The theoretical porosity of the scaffolds was calculated based on the average values for fiber diameter (*d*_1_), fiber spacing (*d*_2_), and layer thickness (*d*_3_), measured by ImageJ software and reported in Table S-4. As shown, the experimental porosities were comparable to the calculated theoretical ones. However, the experimental porosity was slightly lower than the theoretical one, which could be due to the minor accumulation of the polymer on the surrounding area of the scaffolds. The 3D printed structures looked homogeneous and reproducible over the layers, as confirmed by the low standard deviation of *d*_1_, *d*_2_, and *d*_3_ parameters. Handling of scaffolds did not cause any delamination, suggesting adequate fusion of the filaments. To assess the effectiveness of the fusion, mechanical tests were performed, which are discussed in the next section.

In order to study the effect of the 3D printing process on the crystalline morphology of the PEAs, polarized optical microscopy (POM; BX53 Olympus with a DP26 camera, Japan) was applied. To prepare the sample for POM, very thin cross-section layers (about 5 μm) of the filaments, taken from the PEA 3D printed scaffolds, were cut using a Leica EM UC7 ultramicrotome. A representative POM micrograph (Figure S-11) illustrated a granular texture of the microspherulitic morphology for PEA-MM_w_ 3D printed sample, which was homogeneously distributed from the surface to the center of a filament’s cross section. These results next to the DSC measurements of the 3D printed PEAs first heating cycle (Table 4) further confirmed that the 3D printed PEAs preserve their semicrystalline structure due to their fast crystallization rate, despite the very fast cooling rate during the FMD process. However, by comparing the Δ*H*_m_ values in Tables 2 and 4, there is no doubt that the 3D printed polymers exhibit a lower crystallinity degree than the original polymers due to a faster cooling rate.

### 5 Mechanical Characterization

**Figure 7:**
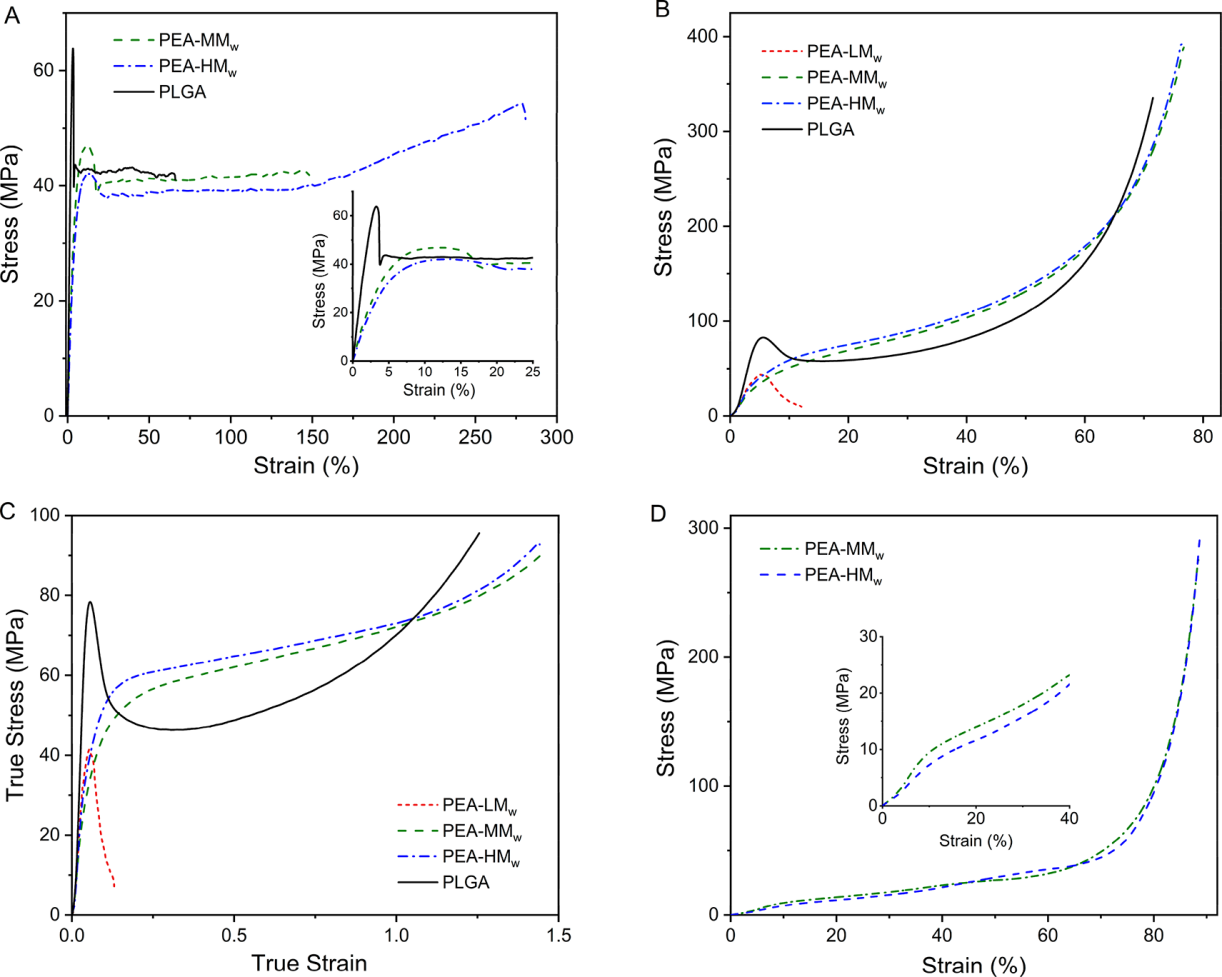
Tensile strength graphs of the PEAs vs PLGA (A); Compression test of the bulk cylindrical samples of PEAs vs PLGA, engineering stress–strain graphs (B) and true stress–strain graphs (C) and the engineering stress–strain graphs for the mechanical compression test of the PEAs 3D printed scaffolds (D).

Tensile tests of PEA-MM_w_, PEA-HM_w_, and PLGA were performed on dog bone samples punched from compression molded films. The films of PEA-LM_w_ were brittle and consequently not suitable for the tensile measurements. According to the GPC results taken from the samples after preparation via the compression molding, all of the PEAs and PLGA samples were relatively stable upon processing since only small molecular weight changes were observed (Table S-2). As observed from the stress–strain curves in Figure 7A and the derived mechanical parameters in Table S-5, the E-modulus of PLGA was significantly higher than those of the PEA2 and PEA3, 2.42 versus 1.02 and 0.9, respectively. The difference in E-modulus is explained by the fact that, at the temperature of testing (room temperature), PLGA was below its glass transition temperature (57 °C), while the PEAs were just above the glass transition temperature where segmental conformational motion lowers the resistance against deformation and thus the E-modulus.^54^ Similarly, the difference in glass transition temperature related to the temperature of mechanical testing explains the differences in yields stress, which is considered the mechanical equivalent of the glass transition temperature and thus the stress needed to induce conformational and translational motion that lead to flow.^52^ Due to the absence of segmental conformational and translational motion in PLG8218, a higher stress level was needed to induce flow; 65.5 MPa in comparison to 46.7 and 42.2 for PEA-MM_w_ and PEA-HM_w_, respectively. The fact that both the E-modulus and yield stress of PEA-MM_w_ was higher than for PEA-HM_w_ can be explained by the difference in crystallinity. The enthalpy of melting of PEA-HM_w_ in the first heating cycle, 58.7 J/g, was distinctly lower than PEA-MM_w_, 71.7 J/g (Table S-5). Such a difference is likely caused by the differences in molar mass, where depending on the cooling rate a higher molar mass may reduce the crystallization rate.^51^

The engineering stress–strain (based on the original cross-sectional area of the material) and true stress–strain (based on the material’s instantaneous cross-section during the test) graphs of the PEAs and PLGA bulk cylinders for the compression test are illustrated in Figure 7B and C, respectively. Since stress delocalization hinders the direct mechanical comparison of the polymeric samples and prevents the revealing of the physical origin of the differences of post-yield deformation engineering such as toughness, true stress–true strain curves were determined in compression testing. PLGA exhibited a similar tensile test behavior with showing higher elastic modulus and yield stress values compared with the PEA samples. Among the PEAs, PEA-LM_w_, despite its lower *M*_w_, showed a relatively higher elastic modulus than the other PEAs due to the higher crystallinity, as shown by the higher melting enthalpy in the first DSC heating cycle (Table 3). The first heating of the cylindrical samples provides information on the structure tested and thus responsible for the behavior in mechanical testing. However, PEA-LM_w_ showed a stiff behavior with the lowest stress values among the PEA samples, and the cylinders collapsed before the compression force reached to its higher values, likely due to the absence of an adequate entanglement network.^52^ Besides the identical trends in terms of E-modulus and yield stress observed in the tensile experiments, albeit minor variations, due to similar crystallinity of PEA-MM_w_ and PEA-HM_w_ cylinders (Table 3), all samples except PEA-LM_w_ possessed strain hardening at high strain. Strain hardening, a relatively high tensile strength at break coupled with reasonable ductility, makes these PEAs an advantageous class of biomaterials to serve in the field of tissue engineering.^55^

**Table 3:** Compression Mechanical Test Properties of PEAs and PLGA Bulk Cylinders

sample	diameter (mm)	height (mm)	density (g/cm^3^)	Δ*H*_m_^a^ (J/g)	*E*_mod_ (eng; GPa)	σ_yield_ (eng; MPa)	σ_max_ (MPa)	ε_break_ (%)	*E*_mod_ (true; GPa)	σ_yield_ (true; MPa)
PEA-LM_w_	8.05 ± 0.05	6.8 ± 1.0	1.24 ± 0.008	79.5	1.44 ± 0.07	44.4 ± 2.5	44.4 ± 2.5	13.2 ± 2.9	1.33 ± 0.06	41.8 ± 2.2
PEA-MM_w_	7.98 ± 0.06	5.57 ± 0.36	1.21 ± 0.010	72.3	1.01 ± 0.02	58.6 ± 2.8	410.6 ± 28.6	77.4 ± 1.0	0.95 ± 0.02	55.8 ± 2.7
PEA-HM_w_	7.96 ± 0.02	6.35 ± 0.84	1.23 ± 0.004	68.9	1.37 ± 0.11	66.3 ± 2.9	392.2 ± 10.1	76.3 ± 0.3	1.28 ± 0.10	61.0 ± 1.3
PLGA	8.13 ± 0.01	5.95 ± 0.42	1.28 ± 0.004		2.69 ± 0.09	83.8 ± 1.1	325.0 ± 29.2	71.6 ± 0.8	2.51 ± 0.08	79.0 ± 1.3

a DSC data of the bulk cylinders were acquired from their first cycle. Measurements were performed under nitrogen flow, heating and cooling rate: 10 °C/min.

The compression mechanical test results of the 3D printed scaffolds for PEA-MM_w_ and PEA-HM_w_ showed that despite the difference in their *M*_w_, most of the engineering stress–strain values of these two samples were comparable (Figure 7D and Table 4). Nonetheless, PEA-MM_w_ showed a relatively higher elastic modulus. It seems that the higher crystallinity of PEA-MM_w_ and the higher *M*_w_ of PEA-HM_w_ played a role in their final mechanical properties. In the compression test of the bulk cylinders, as the crystallinity of these samples are in a similar range due to the low cooling rate of the polymer melts during sample preparation, it seems that the higher *M*_w_ of PEA-HM_w_ led to its higher *E*_mod_ compared with PEA-MM_w_. However, for the 3D printed samples, as the cooling rate during the 3D printing of scaffolds is relatively high, the increase in the molecular weight of PEA-HM_w_ produced relatively lower crystallinity degrees associated with the molecular diffusion problems connected to their longer chains.^51^ The melt viscosity of the molten polymers scales with molecular weight (above the critical molecular weight for the development of entanglements), and therefore, higher molecular weights are well-known to cause reductions in diffusion coefficients and thus interfacial bonding.^56^ Furthermore, due to the higher cooling rates in FDM, differences in the crystallinity of the scaffolds are observed according to the Δ*H*_m_ value of the 3D printed PEAs measured from their first heating cycle (Table 4). The differences in crystallinity were in a similar order of magnitude as in tensile testing where the modulus was only slightly affected. Hence, the higher elastic modulus of PEA-MM_w_, 134.7 in comparison to 93.0 MPa for PEA-HM_w_, is likely caused by higher crystallinity and mechanically more effective interfacial bonding.

**Table 4:** Compression Mechanical Test Properties for the 3D Scaffolds

sample	diameter (cm)	height (cm)	porosity (exp; %)	Δ*H*_m_^a^ (J/g)	*E*_mod_ (MPa)	σ_yield_ (MPa)	σ_max_ (MPa)	ε_break_ (%)
PEA-MM_w_	8.11 ± 0.07	8.58 ± 0.02	50.0 ± 0.9	68.8	134.7 ± 12.5	9.5 ± 0.7	274.0 ± 9.1	88.4 ± 0.13
PEA-HM_w_	7.91 ± 0.17	8.69 ± 0.09	49.6 ± 4.3	55.7	93.0 ± 8.6	7.4 ± 0.7	289.5 ± 13.1	89.0 ± 0.33

a DSC data of the 3D scaffolds were acquired from their first cycle. Measurements were performed under nitrogen flow, heating and cooling rate: 10 °C/min.

### 6 In Vitro Evaluation

**Figure 8:**
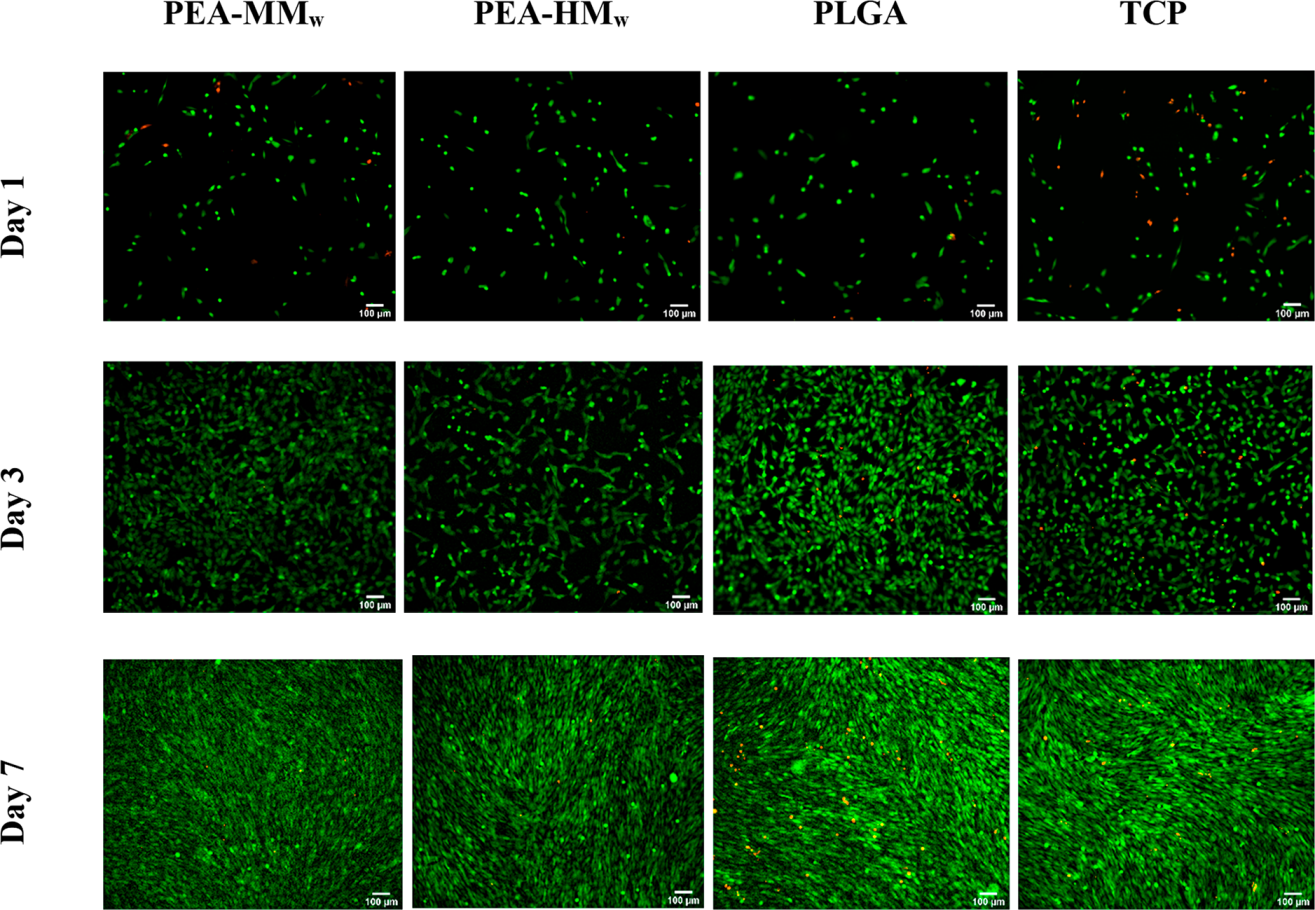
Fluorescent microscopy images of MG63 osteosarcoma cells seeded on different polymers after 1, 3, and 7 days. The live cells are shown in green and the dead cells are shown in red.

MG63 cell viability, attachment, spreading, and proliferation on the PEAs was examined in comparison with a biomedical grade PLGA to evaluate whether or not PEAs are suitable materials for tissue engineering applications. This was done using TCP, a cell culturing material based on polystyrene, which was used as a golden standard control. Figure 8 shows the live/dead assay of the samples with living cells labeled in green and dead cells in red. The vast majority of the cells on the tested samples were viable at all of the time points and both of the PEAs seemed comparable with PLGA and TCP. The cells looked well distributed and attached at day 1 and the majority of the cells were alive. The cell number significantly increased at day 3 and more cells with healthy osteoblastic spindle shape could be observed, which this was a good indication of the cell-adherent properties of the synthesized materials. Furthermore, proliferating cells showed clusters, which could indicate good cell–cell signaling. At day 7, there was almost no rounded-shape cells and cell confluency was reached, as shown by the whole surface of the films being covered. This demonstrated the promising biocompatibility of the tested PEAs, next to the biomedical grade PLGA, providing a suitable environment for MG63 cell growth.

**Figure 9:**
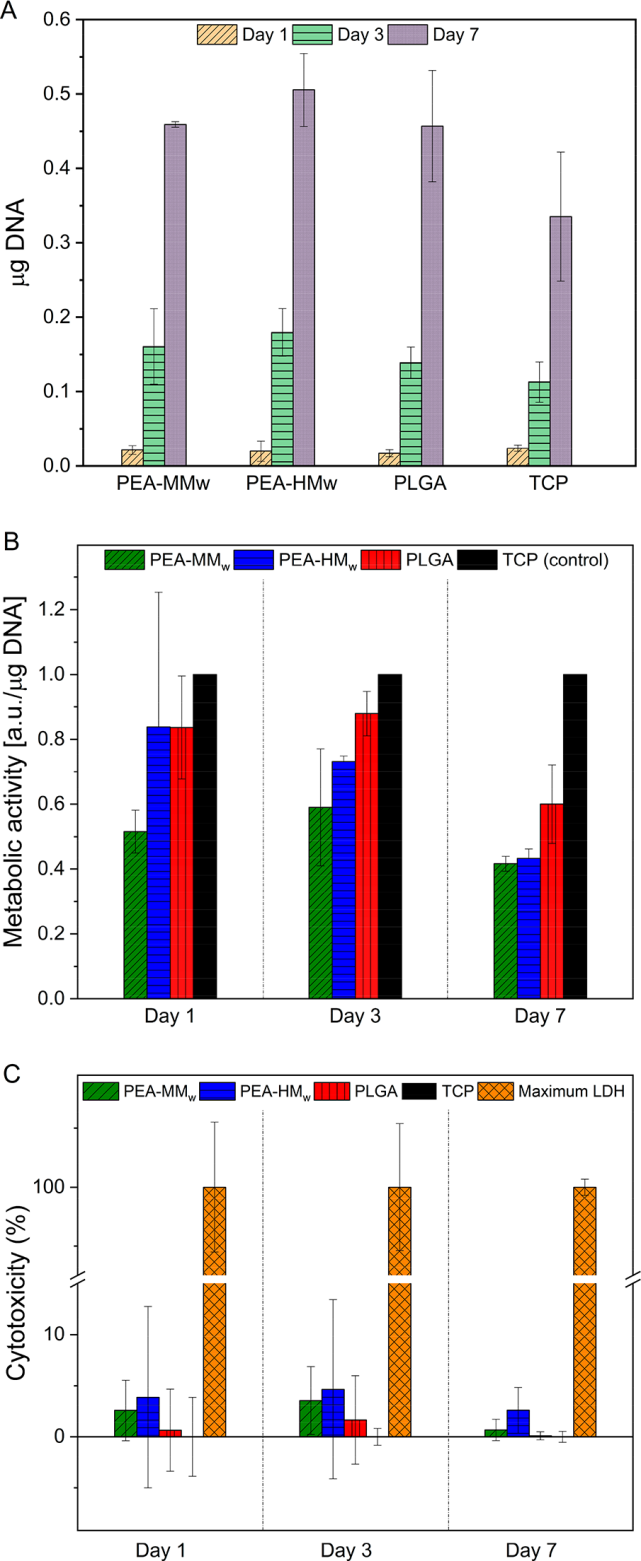
DNA quantification of the MG63 cells seeded on different polymer films after 1, 3, and 7 days of culture (A); The metabolic activity (normalized to DNA content) of MG63 cells seeded on polymers at different time points (B) and LDH release of the MG63 cells seeded on different samples at days 1, 3, and 7 as an indication of the cytotoxicity of the polymers to the cells (the maximum cytotoxicity is shown based on the maximum LDH release of the cells seeded on TCP as positive control) (C).

Quantitative analysis was also in agreement with the live/dead assay. As shown in Figure 9A, the DNA quantification showed a similar cell number at all the time points, with an increase in cells with increasing culturing time. The metabolic activity of the PEAs compared with a biomedical grade PLGA and TCP is shown in Figure 9B. The metabolic activity values for each of the samples are normalized to their corresponding μg DNA quantified at any of the time points. For an easier comparison, all of the DNA normalized metabolic activities were divided by the average metabolic activity of the TCP for that time point. TCP is expected to support better cell adhesion and proliferation as a golden standard. Hence, it was also to be expected that TCP showed higher metabolic activity compared with the other samples. The cells seeded on the PEAs showed metabolic activities comparable with PLGA. At day 7, the difference between the metabolic activities of the cells seeded on the TCP samples compared to the other samples was higher. This lower metabolic activity of PLGA and PEAs compared with TCP could be due to the relatively higher population of the cells on the film surface according to the DNA quantification results. The limited space for the cells to grow at day 7 could lead to a reduction in their proliferation activity, as similar results have been reported in other works.^57,58^

The LDH cytotoxicity assay for the samples was also performed in order to evaluate the potential cytotoxicity of the PEAs to the living cells in comparison with PLGA and TCP (Figure 9C). Not surprisingly, TCP showed almost no cytotoxicity. All of the samples showed low cytotoxicity at all of time points with no significant differences. Both of the PEAs showed average cytotoxicity below 5% further indicating the biocompatibility of the synthesized PEAs for biomedical application. PEA-MM_w_ and PLGA showed very low cytotoxicity and were quite comparable to each other. PEA-HM_w_ also showed relatively low cytotoxicity, which was slightly higher than PEA-MM_w_.

#### Bioactivity Evaluation Using SBF

One of the challenges in the use of artificial materials for bone tissue engineering has been the isolation of the materials from the surrounding bone defects. It has been shown that the materials with the ability to form bonelike apatite on their surface, when implanted, would be able to solve this isolation problem as they bond to the living bone tissue through the apatite layer. The evaluation of the ability of the materials for the formation of bonelike apatite is important, as it will be helpful for the prediction of the in vivo bone bioactivity of the material and it reduces the number of animals needed during the experiments.^59^ The ability for the formation of bonelike apatite has been studied for different materials using a simulated body fluid (SBF) containing the essential ions for the formation of hydroxyapatite. However, the concentrations of the ions in many studies are much higher than the concentrations of those of the human blood plasma. Therefore, for a valid evaluation, the concentration of the ions in SBF needs to be nearly similar to those of the human blood plasma.^46,59^ The current work investigates the ability of the formation of bonelike apatite on the surface of PEAs and PLGA using SBF (1×), which includes the ions in physiological concentration similar to real body fluid. The development of tailor-made biomaterials providing different mechanical properties with bone-bonding capability is of high importance in tissue engineering. Therefore, testing the bioactivity of the PEAs using SBF next to their cell–polymer interactions will provide valuable information regarding their potentials for biomedical applications.

**Figure 10:**
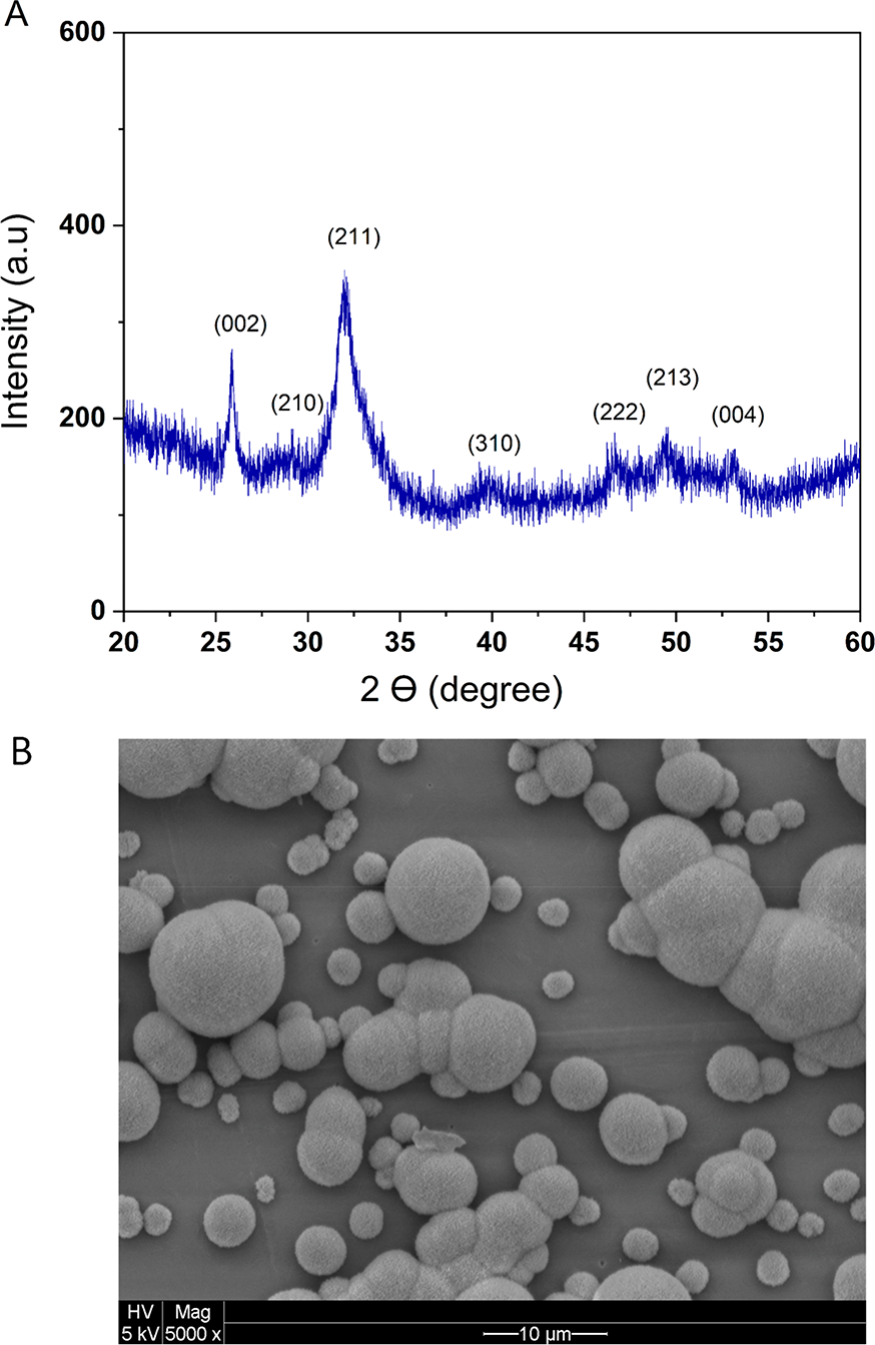
XRD pattern (A) and SEM image (B) of the bonelike hydroxyapatite formed on the surface of PEA-MM_w_.

The ability of the formation of bonelike hydroxyapatite (HA) on the surface of PEA-MM_w_ and PLGA as an important factor for the bioactivity of the materials was studied through soaking the polymers films in SBF (1×). The SEM images of the samples clearly showed the mineralization on the surface of PEA-MM_w_ and PLGA. The XRD analysis of the white powder collected from the surface of the films confirmed the formation of bonelike HA on the surface of PEA-MM_w_ (Figure 10A). The XRD pattern obtained was in accordance with the patterns of the hydroxyapatite of human bone and also the nanocrystalline HA.^60,61^ The SEM image of the surface of PEA-MM_w_ indicated the round shape and spongy morphology of the mineralized hydroxyapatite on its surface (Figure S-12). A representative SEM image of the mineralization on the surface of PEA-MM_w_ after 14 days is shown in Figure 10B. The SEM images of the PEAs and PLGA films during the incubation time revealed that the amounts of hydroxyapatite formed on the surface of the films increased over the time. In addition, as shown in Figure S-12, the surface of PLGA films were covered with the mineralized HA faster than those of PEA-MM_w_. This could be due to the formation of more COO^®^ groups on the surface of PLGA upon the hydrolysis of ester bonds during the time compared with PEA-MM_w_. The presence of a higher number of negatively charged COO^®^ groups on the surface of PLGA could further induce the nucleation and formation of HA as a result of complexation with Ca^2+^ ions.^62^ However, as illustrated in Figure S-12, the undesired delamination defects of hydroxyapatite layers from the polymer’s surface observed in PLGA was considerably higher compared with PEA-MM_w_. On the other hand, more round-shaped HA with a spongy morphology and a larger particle size was observed on the surface of PEA-MM_w_ compared with PLGA. This could be due to the slower mineralization of HA, which could provide more time for the growth and better bonding of the crystals on the surface of PEA-MM_w_ compared with PLGA. Nonetheless, the effects of different morphology of HA on the cells growth and proliferations need to be further investigated. The SEM images of HA mineralization on the surface of the 3D printed PEA-MM_w_ (Figure S-13) showed that the mineralization was homogeneously achieved on the curved surface of the filaments with no major delamination problem. Furthermore, the mineralization speed on the surface of the 3D printed PEA-MM_w_ seemed to be faster than their corresponding films. This could be due to the higher surface area of the 3D printed samples compared to the films, which could further promote the formation of HA.^46^

## Conclusion

α-Amino acid-based PEAs as a class of polymers with unique properties were successfully synthesized. The potential of PEAs in AM for biomedical applications was evaluated. The synthesized PEAs revealed different mechanical properties, which were dependent on the molecular weights and their corresponding crystallization rates and final crystallinity. As targeted, the thermomechanical properties were marked by a *T*_g_ just below body temperature and ductility, which is advantageous in biomedical engineering. DSC analysis revealed high crystallization rates of the PEAs. The rheology studies provided information on the need for end-capping of the PEAs, which could allow for facile and reproducible extrudability. Indeed, the non-end-capped PEAs cross-linked during the melt conditions and therefore challenged the steadiness of the procedure over time. The end-capped PEA-MM_w_ and PEA-HM_w_ proved to be suitable for AM, as they were processed under steady conditions with good shape retention and fusion between the layers.

The biocompatibility assessment of the PEA films showed good cell–material interactions and low cytotoxicity levels comparable with the biomedical grade poly(lactide-*co*-glycolide) PLGA, thus, providing a suitable environment for cell attachment, spread, and proliferation. In addition, the mineralization of bonelike HA on the surface of PEA films and 3D printed scaffolds using SBF further indicated their bioactivity. This suggests that the PEAs with good cytocompatibility, proper printability, and good mechanical properties can serve as promising candidates for AM of 3D scaffolds in tissue-engineering applications. Furthermore, the active solution polycondensation method can provide a versatile procedure for the synthesis of tailor-made PEAs with different physiochemical properties via purposefully designed monomers according to the target properties. The current work offers a platform for future developments in AM of α-amino acid-based PEAs, which provides further opportunities in the field of tissue engineering.

## Supporting Information Available

^1^H and ^13^C NMR spectra of the synthesized monomers and polymers, shape and dimension of the specimens prepared and used for the mechanical tests, TGA and DSC analysis of the PEAs and PLGA, polarized optical micrograph of the cross-section of PEA-MM_w_ 3D printed scaffold, SEM images of the mineralization of hydroxyapatite (HA) on the surface of PEA-MM_w_ films and PLGA films and PEA-MM_w_ 3D printed scaffolds after exposure to simulated body fluid (SBF), the SBF solution composition, GPC analysis of the PEAs and PLGA after different thermal processing conditions, 3D printing fabrication parameters, porosities of the PEAs 3D printed scaffolds, tensile strength properties of PEAs and PLGA (DOCX)
