# Supplementary material for: Additive Manufacturing of α-Amino Acid Based Poly(ester amide)s for Biomedical Applications
Source: Biomacromolecules. 2022 Jan 20;23(3):1083–100. doi: 10.1021/acs.biomac.1c01417 (PMC8924872; doi:10.1021/acs.biomac.1c01417)
Supplement: Supplementary file 1 — bm1c01417_si_001.docx [file bm1c01417_si_001.docx]

**Supporting information**

Additive manufacturing of α-amino acid based Poly(ester amide)s for biomedical applications

^†,‡^Vahid Ansari, ^†,‡^Andrea Calore, ^†^Jip Zonderland, ^‡^Jules A.W. Harings*, ^†^Lorenzo Moroni* ^‡^Katrien V. Bernaerts*

^†^Complex Tissue Regeneration Department, MERLN Institute for Technology Inspired Regenerative Medicine, Maastricht University, Universiteitssingel 40, 6229 ER, the Netherlands

^‡^Aachen-Maastricht Institute for Biobased Materials (AMIBM), Maastricht University, P.O. Box 616, 6200MD Maastricht, The Netherlands

*Email: [katrien.bernaerts@maastrichtuniversity.nl](mailto:katrien.bernaerts@maastrichtuniversity.nl), l.moroni@maastrichtuniversity.nl, jules.harings@maastrichtuniversity.nl

**Figure S-1.** ^1^H-NMR spectrum of di-p-nitrophenyl adipate (monomer A)

**Figure S-2.** ^13^C-NMR spectrum of di-p-nitrophenyl adipate (monomer A)

**Figure S-3.** ^1^H-NMR spectrum of di-p-toluenesulfonic acid salt of bis (glycine)-hexane 1,6-diester (monomer B)

**Figure S-4.** ^13^C-NMR spectrum of di-p-toluenesulfonic acid salt of bis (glycine)-hexane 1,6-diester (monomer B)

**Figure S-5.** The shape and dimension of the different specimens prepared for the mechanical tests. A dog bone prepared from the polymer films using the cutting device (ISO 527-2 1BB) (A), a cylindrical shaped 3D printed PEA (B), a bulk cylinder made of PEA (C), a bulk cylinder covered by PTFE sheets and a droplet of surfactant on the top and bottom of the PTFE sheets before running the compression test (D) and the stainless steel mold used for the preparation of the bulk cylinders via compression molding (E).

**Figure S-6.** ^1^H-NMR spectrum of PEA-HM_w_ in DMF-d_7_. The amount of residual DMSO in the sample was calculated according to the ratio of the integrals of DMSO peak appeared at 2.59 ppm (attributed to six protons) and the peak d at 2.25 ppm (attributed to two protons of CH_2_ group) of the PEA’s repeating unit. The weight of the PEA’s repeating unit (342.41 g/mol) and DMSO (78.13 g/mol) were used in the following formula to calculate the weight percentage of DMSO presenting in the sample: wt. % DMSO = [(0.08/6) mol DMSO / (1/2) mol PEA × (78.13 g/mol DMSO) / 342.41 g/mol PEA)] × 100 = 0.61 %.

**Figure S-7.** ^13^C-NMR spectrum of PEA-LM_w_


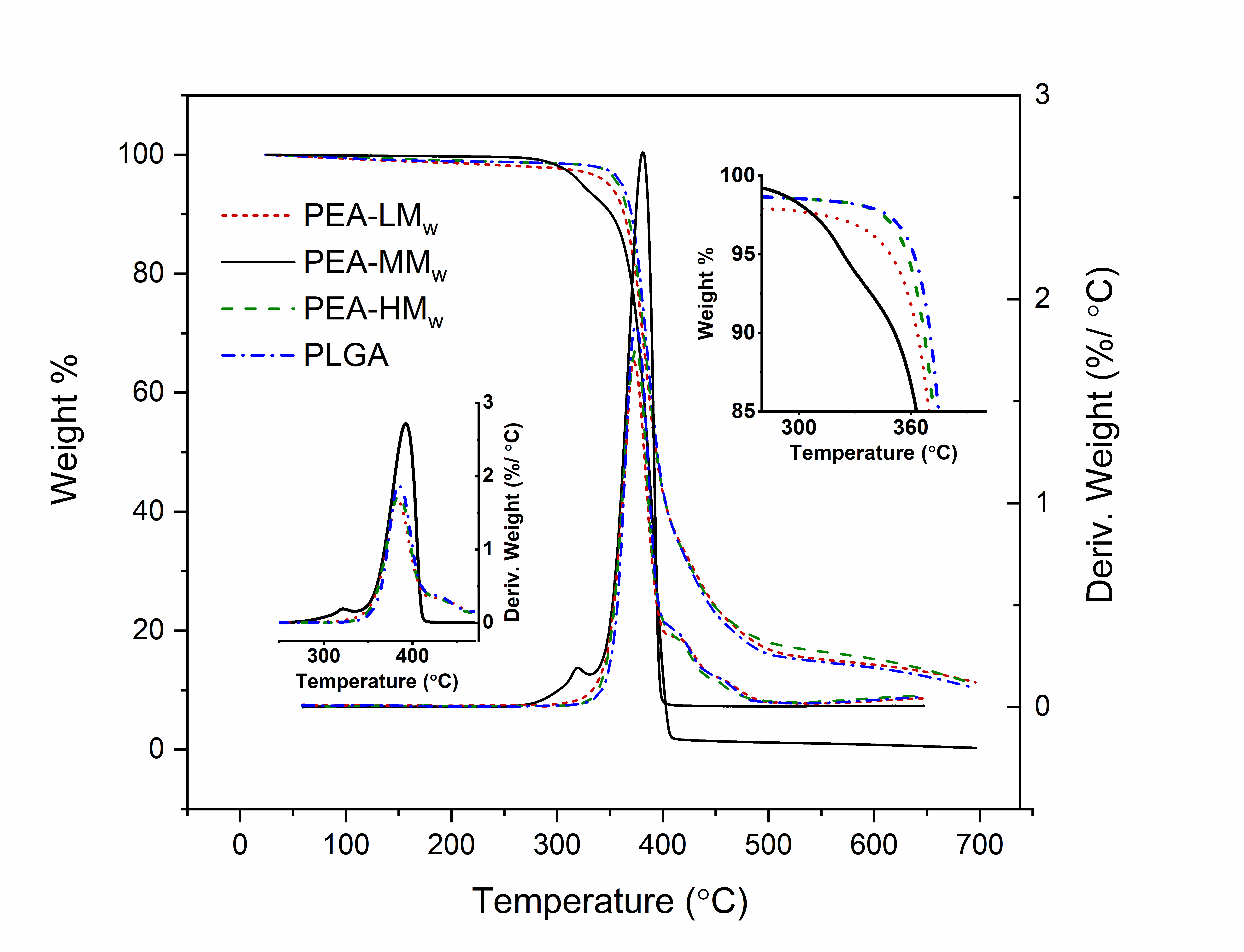


**Figure S-8.** TGA curves of the end-capped PEAs in comparison with PLGA


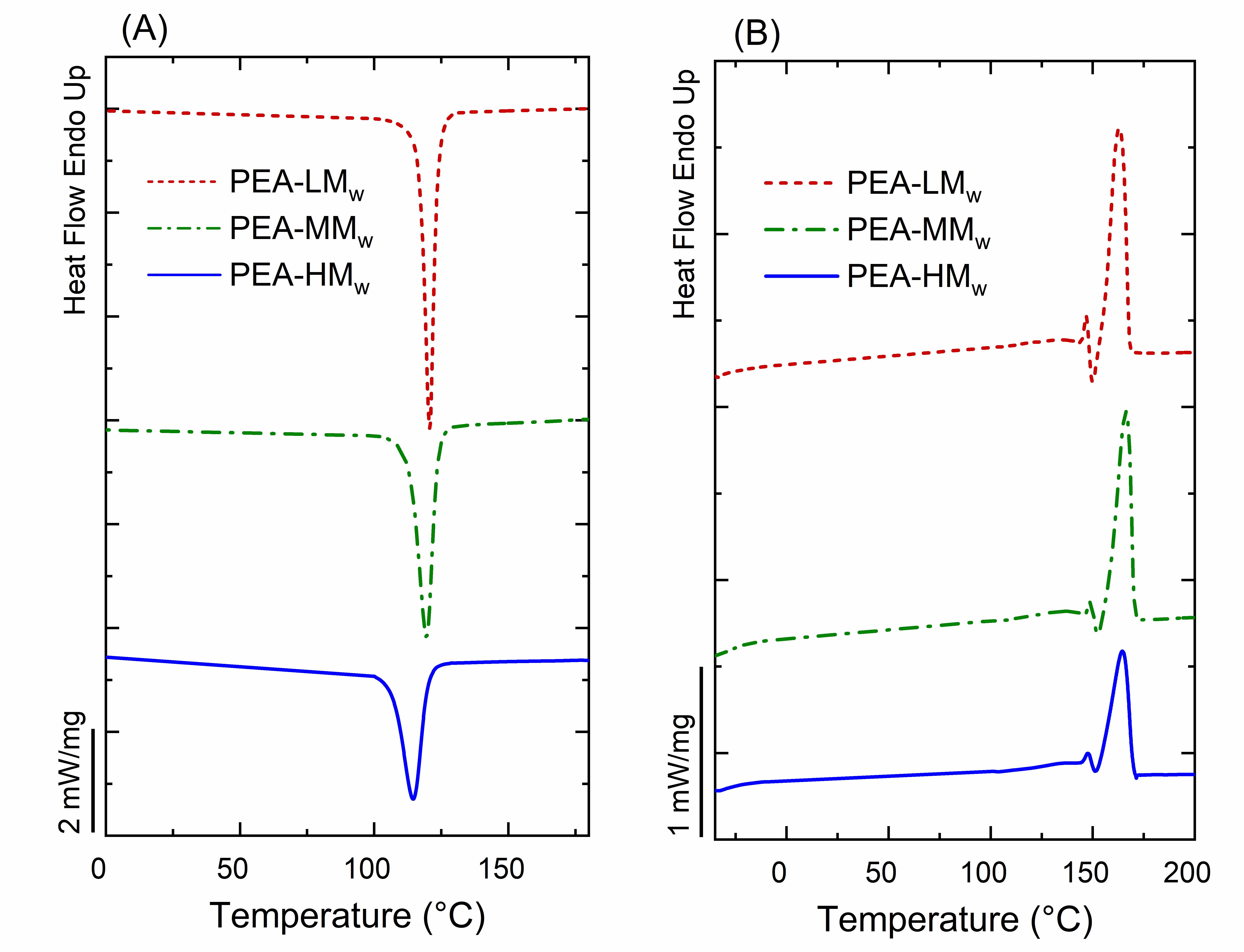


**Figure S-9.** DSC thermograms of PEAs with different molecular weights, cooling with a rate of 30 °C/min (A) and second heating with a rate of 10 °C/min (B).


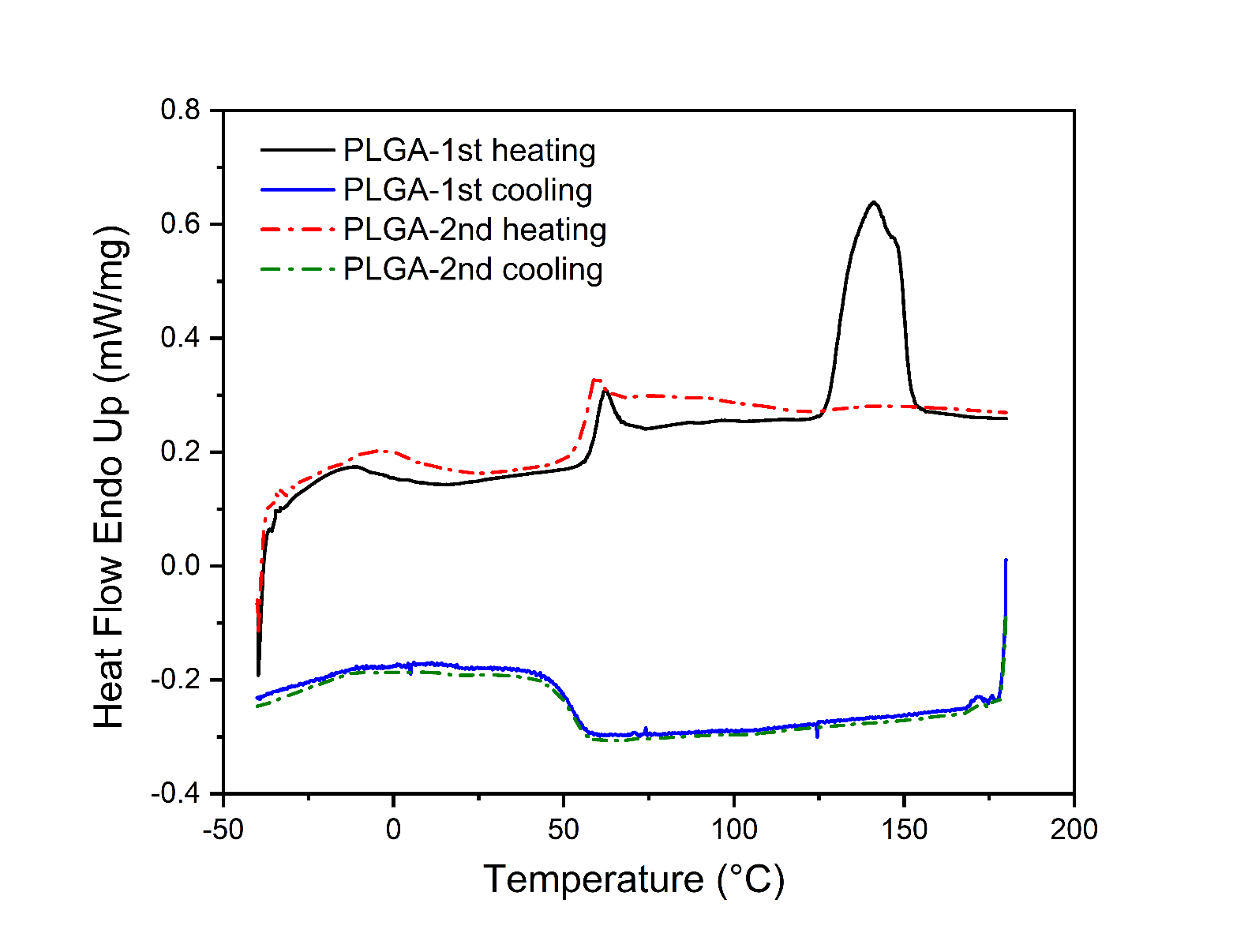


**Figure S-10.** DSC of PLGA (heating and cooling rate 10 °C/min).


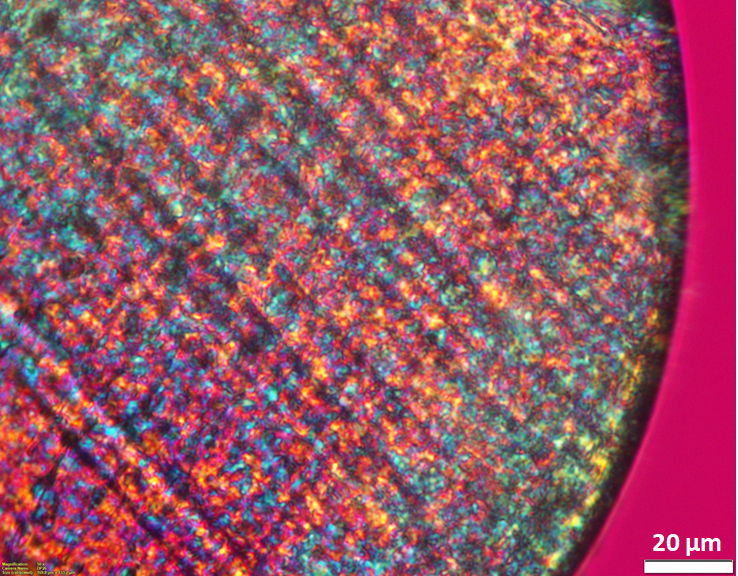


**Figure S-11.** Polarized optical micrograph of the cross-section of a filament taken from a PEAs 3D printed scaffold (PEA-MM_w_).

**Figure S-12.** SEM images (with different magnifications) of the mineralization of hydroxyapatite on the surface of PEA-MM_w_ and PLGA films after exposure to the SBF at different time points.

**Figure S-13.** SEM images (with different magnifications) of the mineralization of hydroxyapatite on the surface of 3D printed scaffolds made of PEA-MM_w_ at different time points.

**Table S-1.** The composition of the solutions A and B used for the preparation of SBF (1x).

| **Materials** | **Purity**  **(%)** | **Solution A**  **(g/L)** | **Solution B**  **(g/L)** |
| --- | --- | --- | --- |
| **NaCl** | 99.5 | 6.129 | 6.129 |
| **NaHCO_3_** | 99.5 | 5.890 | - |
| **Na_2_HPO_4_.2H_2_O** | 99.0 | 0.498 | - |
| **CaCl_2_** | 95.0 | - | 0.540 |

**Table S-2.** The GPC analysis of different polymers after being processed for sample preparation using different techniques in comparison with their original M_w_.

| **Sample** | **Original** | | **After rheology**  **(frequency sweeps)** | | **Films preparation**  **for tensile test** | | **Bulk cylinders** | | **3D printed scaffolds** | |
| --- | --- | --- | --- | --- | --- | --- | --- | --- | --- | --- |
|  | ***M*_w_ ^a^**  **(g/mol)** | ***Ð*** | ***M*_w_ (g/mol)** | ***Ð*** | ***M*_w_ (g/mol)** | ***Ð*** | ***M*_w_ (g/mol)** | ***Ð*** | ***M*_w_ (g/mol)** | ***Ð*** |
| **PEA-LM_w_** | 21800 | 1.9 | 41300 | 2.8 | - | - | - | - | - | - |
| **PEA-LM_w_-Endcapped** | 24700 | 1.9 | 28900 | 2.3 | 23600 | 2.0 | 24400 | 1.9 | 25500 | 1.9 |
| **PEA-MM_w_** | 52900 | 2.0 | 84500 | 2.5 | - | - | - | - | - | - |
| **PEA-MM_w_-Endcapped** | 60100 | 2.0 | 66400 | 2.2 | 54100 | 2.0 | 62400 | 2.1 | 66700 | 2.1 |
| **PEA- HM_w_** | 101500 | 2.4 | - ^b^ | - | - | - | - | - | - | - |
| **PEA-HM_w_ -Endcapped** | 122400 | 2.4 | 106200 | 2.7 | 116800 | 2.4 | 117100 | 2.4 | 109100 | 2.5 |
| **PLGA** | 209300 | 2.3 | 212100 | 2.3 | 187700 | 2.3 | 189400 | 2.3 | - | - |

^a^ GPC in HFIP/0.19% NaTFA, RI detection.

^b^ The sample swelled in HFIP and did not dissolve due to the crosslinking.

**Table S-3.** Fabrication parameters used for 3D printing of PEAs.

| Sample | N_2_ pressure (bar) | Temperature (°C) | Screw speed (R/s) | Feed speed (XY)  (mm/min) |
| --- | --- | --- | --- | --- |
| PEA-MM_w_ | 7.3 | 200 | 50 | 500 |
| PEA-HM_w_ | 10 | 200 | 50 | 300 |

**Table S-4.** Porosities of 3D printed PEA scaffolds.

| Sample | Experimental porosity  (%) | Theoretical porosity (%) | Fiber diameter  (mm) | Fiber spacing (mm) | Layer thickness  (mm) |
| --- | --- | --- | --- | --- | --- |
| PEA-MM_w_ | 50.0 ± 0.9 | 51.4 | 0.44 ± 0.01 | 0.82 ± 0.01 | 0.39 ± 0.01 |
| PEA-HM_w_ | 49.6 ± 4.3 | 52.7 | 0.42 ± 0.01 | 0.80 ± 0.01 | 0.37 ± 0.01 |

**Table S-5.** The tensile strength properties of the PEAs and PLGA.

| Sample | Thickness  (µm) | Δ*H*_m_^a^  (J/g) | E_Mod_  (GPa) | σ_Yield_  (MPa) | ε_Yield_  (%) | σ_Max_  (MPa) | σ_Break_  (GPa) | ε_Break_  (%) |
| --- | --- | --- | --- | --- | --- | --- | --- | --- |
| PEA-MM_w_ | 215 ± 5 | 71.7 | 1.02 ± 0.04 | 46.7 ± 0.7 | 12.5 ± 0.4 | 46.7 ± 0.7 | 28.0 ± 8.7 | 143.8 ± 38.5 |
| PEA-HM_w_ | 214 ± 8 | 58.7 | 0.90 ± 0.05 | 42.2 ± 1.5 | 13.2 ± 0.6 | 52.9 ± 2.8 | 43.6 ± 10.3 | 272.2 ± 15.6 |
| PLGA | 224 ± 8 | - | 2.42 ± 0.07 | 65.5 ± 5.6 | 3.3 ± 0.06 | 65.5 ± 5.6 | 39.0 ± 1.4 | 58.6 ± 9.5 |

^a^ DSC data of the tensile bars were acquired from their first cycle. Measurements were performed under nitrogen flow, heating and cooling rate: 10 °C/min.
